# Prime-seq, efficient and powerful bulk RNA sequencing

**DOI:** 10.1186/s13059-022-02660-8

**Published:** 2022-03-31

**Authors:** Aleksandar Janjic, Lucas E. Wange, Johannes W. Bagnoli, Johanna Geuder, Phong Nguyen, Daniel Richter, Beate Vieth, Binje Vick, Irmela Jeremias, Christoph Ziegenhain, Ines Hellmann, Wolfgang Enard

**Affiliations:** 1 Anthropology & Human Genomics, Faculty of Biology, Ludwig-Maximilians University, Großhaderner Str. 2, 82152 Martinsried, Germany; 2 Graduate School of Systemic Neurosciences, Faculty of Biology, Ludwig-Maximilians University, Martinsried, Germany; 3 Research Unit Apoptosis in Hematopoietic Stem Cells, Helmholtz Zentrum München, German Research Center for Environmental Health (HMGU), Munich, Germany; 4 German Cancer Consortium (DKTK), Partner Site Munich, Munich, Germany; 5 Department of Pediatrics, Dr. von Hauner Children’s Hospital, Ludwig-Maximilians University, Munich, Germany; 6 Department of Cell and Molecular Biology, Karolinska Institutet, Stockholm, Sweden

**Keywords:** RNA-seq, Transcriptomics, Genomics, Power analysis

## Abstract

Cost-efficient library generation by early barcoding has been central in propelling single-cell RNA sequencing. Here, we optimize and validate prime-seq, an early barcoding bulk RNA-seq method. We show that it performs equivalently to TruSeq, a standard bulk RNA-seq method, but is fourfold more cost-efficient due to almost 50-fold cheaper library costs. We also validate a direct RNA isolation step, show that intronic reads are derived from RNA, and compare cost-efficiencies of available protocols. We conclude that prime-seq is currently one of the best options to set up an early barcoding bulk RNA-seq protocol from which many labs would profit.

**Supplementary Information:**

The online version contains supplementary material available at 10.1186/s13059-022-02660-8.

## Background

RNA sequencing (RNA-seq) has become a central method in biology and many technological variants exist that are adapted to different biological questions [1]. Its most frequent application is the quantification of gene expression levels to identify differentially expressed genes, infer regulatory networks, or identify cellular states. This is done on populations of cells (bulk RNA-seq) and increasingly with single-cell or single-nucleus resolution (scRNA-seq). Choosing a suitable RNA-seq method for a particular biological question depends on many aspects, but the number of samples that can be analyzed is almost always a crucial factor. Including more biological replicates increases the power to detect differences and including more sample conditions increases the generalizability of the study. As the limiting factor for the number of samples is often the budget, the costs of an RNA-seq method are an essential parameter for the biological insights that can be gained from a study. Of note, costs need to be viewed in the context of statistical power, i.e., in light of the true and false positive rate of a method [2, 3] and these “normalized” costs can be seen as cost efficiency. On top of reagent costs per sample, aspects like robustness, hands-on time, and setup investments of a method can also be seen as cost factors. Other important factors less directly related to cost efficiency are the number and types of genes that can be detected (complexity), the amount of input material that is needed to detect them (sensitivity), and how well the measured signal reflects the actual transcript concentration (accuracy).

In recent years, technological developments have focused on scRNA-seq due to its exciting possibilities and due to the urgent need to improve its cost efficiency and sensitivity [4–6]. A decisive development for cost efficiency was “early barcoding”, i.e., the integration of sample-specific DNA tags in the primers used during complementary DNA (cDNA) generation [7, 8]. This allows one to pool cDNA for all further library preparation steps, saving time and reagents. However, the cDNA and the barcode need to be sequenced from the same molecule and hence cDNA-tags and not full-length cDNA sequences are generated. An improvement in measurement noise is achieved by integrating a random DNA tag along with the sample barcode, a Unique Molecular Identifier (UMI), that allows identifying PCR duplicates and is especially relevant for the small starting amounts in scRNA-seq [2, 7, 9]. Optimizing reagents and reaction conditions (e.g., [10, 11]) and the efficient generation of small reaction chambers such as microdroplets [12–14], further improved cost efficiency and sensitivity and resulted in the current standard of scRNA-seq, commercialized by 10X Genomics [5].

Despite these exciting developments, bulk RNA-seq is still widely used and—more importantly—still widely useful as it allows for more flexibility in the experimental design that can be advantageous and complementary to scRNA-seq approaches. For example, investigated cell populations might be homogenous enough to justify averaging, single-cell or single-nuclei suspensions might be difficult or impossible to generate, or single-cell or single-nucleus suspension might be biased towards certain cell types. Most trivial, but maybe most crucial, the number of replicates and conditions is limited due to the high costs of scRNA-seq per sample. Furthermore, as more knowledge on cellular and spatial heterogeneity is acquired by scRNA-seq and spatial approaches, bulk RNA-seq profiles can be better interpreted, e.g., by computational deconvolution of the bulk profile [15]. Hence, bulk RNA-seq will remain a central method in biology, despite or even because of the impressive developments from scRNA-seq and spatial transcriptomics. However, bulk RNA-seq libraries are still largely made by isolating and fragmenting mRNA to generate random primed cDNA sequencing libraries. Commercial variants of such protocols, such as TruSeq and NEBNext, can be considered the current standard for bulk RNA-seq methods. This is partly because improvements of sensitivity and cost efficiency were less urgent for bulk RNA-seq as input amounts were often high, overall expenses were dominated by sequencing costs, and *n* = 3 experimental designs have a long tradition in experimental biology [16]. However, input amounts can be a limiting factor, sequencing costs have decreased and will further decrease, and low sample size is a central problem of reproducibility [17, 18]. To address these needs, several protocols have been developed, including targeted approaches [19–21] and genome-wide approaches that leverage the scRNA-seq developments described above [16, 22]. However, given the importance and costs of bulk RNA-seq, even seemingly small changes, e.g., in the sequencing design of libraries [16], the number of PCR cycles [9], or enzymatic reactions [22], can have relevant impacts on cost efficiency, complexity, accuracy, and sensitivity. Furthermore, protocols need to be available to many labs to be useful and insufficient documentation, limited validation, and/or setup costs can prevent their implementation. Accordingly, further developments of bulk RNA-seq protocols are still useful.

**Fig. 1:**
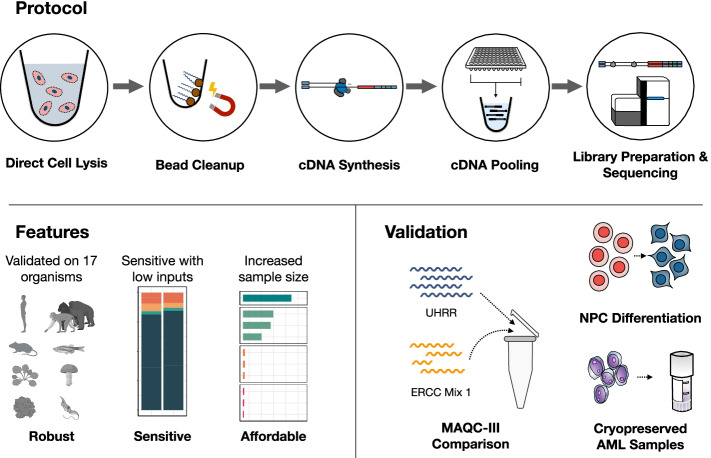
Graphical overview of prime-seq, highlighting its robustness, sensitivity, affordability, and the validation experiments performed. Cells are first lysed, mRNA is then isolated using magnetic beads, and in turn reverse transcribed into cDNA. Following cDNA synthesis, all samples are pooled, libraries are made, and the samples are sequenced. The protocol has been validated on 17 organisms, including human, mouse, zebrafish, and arabidopsis. Additionally, prime-seq is sensitive and works with low inputs, and the affordability of the method allows one to increase sample size to gain more biological insight. To verify prime-seq’s performance, we first compared prime-seq to TruSeq using the publicly available MAQC-III Study data. We then showed robust detection of marker genes in NPC differentiation and high-throughput analysis of AML-PDX patient samples without compromising the archived samples

Here, we have optimized and validated a bulk RNA-seq method that combines several methodological developments from scRNA-seq to generate a very sensitive and cost-efficient bulk RNA-seq method we call prime-seq (Fig. 1, Additional file 1: Fig. S1). In particular, we have integrated and benchmarked a direct lysis and RNA purification step, validated that intronic reads are informative as they are not derived from genomic DNA, and show that prime-seq libraries are similar in complexity and statistical power to TruSeq libraries, but at least fourfold more cost-efficient due to almost 50-fold cheaper library costs. Prime-seq is also robust, as we have used variants of it in 22 publications [9, 23–43], 132 experiments, and in 17 different organisms (Additional file 2: Table S1, Additional file 1: Fig. S2). Additionally, it has low setup costs as it does not require specialized equipment and is well validated and documented. Hence, it will be a very useful protocol for many labs or core facilities that quantify gene expression levels on a regular basis and have no cost-efficient protocol available yet.

## Results

### Development of the prime-seq protocol

**Fig. 2:**
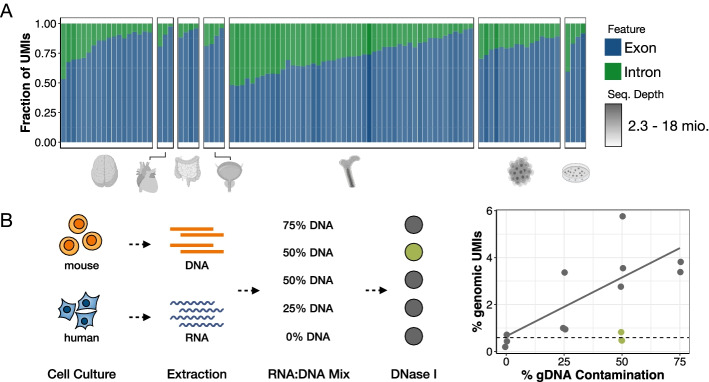
Intronic reads account for a variable but substantial fraction of UMIs and stem from RNA. **A** Fraction of exonic and intronic UMIs from 97 primate and mouse experiments using various tissues (neural, cardiopulmonary, digestive, urinary, immune, cancer, induced pluripotent stem cells). Sequencing depth is indicated by shading of the individual bars. We observe an average of 21% intronic UMIs, with some level of tissue-specific deviations as, e.g., immune cells generally have higher fractions of intronic reads. **B** To determine if intronic reads stem from genomic DNA or mRNA, we extracted DNA from mouse embryonic stem cells (mESCs) and RNA from human-induced pluripotent stem cells (hiPSCs), pooled the two in various ratios (75, 50, 25, and 0% gDNA), and either treated the samples with DNase I (green) or left them untreated (gray). We then counted the percentage of genomic (=mouse-mapped) UMIs. This indicates that DNase I treatment in prime-seq is complete and that observed intronic reads are derived from RNA

The prime-seq protocol is based on the scRNA-seq method SCRB-seq [44] and our optimized derivative mcSCRB-seq [11]. It uses the principles of poly(A) priming, template switching, early barcoding, and UMIs to generate 3′ tagged RNA-seq libraries (Fig. 1 and Additional file 1: Fig. S1). Compared to previous versions as described, e.g., in [32], we have optimized the workflow, switched from a Nextera library preparation protocol to an adjusted version of NEBNext Ultra II FS, and made the sequencing layout analogous to 10X Chromium v3 gene expression libraries to facilitate pooling of libraries on Illumina flow cells, which is of great practical importance [16]. A detailed step-by-step protocol of prime-seq, including all materials and expected results, is available on protocols.io (10.17504/protocols.io.s9veh66). We have so far used this and previous versions of the protocol in 22 publications [9, 23–43] and have generated just within the last year over 24 billion reads from > 4800 RNA-seq libraries in 97 projects from vertebrates (mainly mouse and human), plants, and fungi (Additional file 2: Table S1 and Fig. 2A). From these experiences, we find that the protocol works robustly and detects per sample on average >20,000 genes with 6.7 million reads of which 90.0% map to the genome and 71.6% map to exons and introns (Additional file 2: Table S1). Notably, a large fraction (21%) of all UMIs map to introns with considerable variation among samples (Fig. 2A). Across all data sets, about 8000 genes are detected only by exonic reads, ~ 8000 by exonic and intronic reads, and ~ 4000 by intronic reads only (Additional file 1: Fig. S2B, Additional file 2: Table S1). Previous studies for scRNA-seq data showed that intronic reads can improve cluster identification [45] and allow to infer expression dynamics [46]. Also for bulk RNA-seq data, it has been shown that they are informative [47]. Nevertheless, it is an uncommon practice to use them. This might be due to concerns that intronic reads could at least partially be derived from genomic DNA as MMLV-type reverse transcriptases could prime DNA that escaped a DNase I digest. Therefore, we investigated the origin of the intronic reads in prime-seq.

### Intronic reads are derived from RNA

First, we measured the amount of DNA yield generated from genomic DNA (gDNA). We lysed varying numbers of cultured human embryonic kidney 293T (HEK293T) cells and treated the samples with DNase I, RNase A, or neither prior to cDNA generation using the prime-seq protocol (up to and including the pre-amplification step). Per 1000 HEK cells, this resulted in ~5 ng of “cDNA” generated from gDNA in addition to the 12–32 ng of cDNA generated from RNA (Additional file 1: Fig. S3A). To test the efficiency of DNase I digestion and quantify the actual number of reads generated from gDNA, we mixed mouse DNA and human RNA in different ratios (Fig. 2B). Prime-seq libraries were generated and sequenced from untreated and DNase I-treated samples and reads were mapped to the mouse and human genome (Fig. 2B). In the sample that did not contain any mouse DNA, ~70% of reads mapped to exons or introns (Additional file 1: Fig. S3B) and ~0.5% of the exonic and intronic UMIs mapped to the mouse genome (Additional file 1: Fig. S3C), representing the background level due to mismapping. Importantly, the DNase I-treated sample had almost the same distribution and amount of mismapped UMIs (0.7%), strongly suggesting that the DNase I digest is nearly complete and that essentially all reads in the DNase I-treated sample are derived from RNA (Fig. 2B and Additional file 1: Fig. S3).

As expected, with increasing amounts of mouse DNA, the proportion of mouse-mapped UMIs increased (Fig. 2B), but even with 75% of the sample being mouse DNA, only 3.6% of the UMIs map to the mouse genome, suggesting that also for gDNA-containing samples (e.g., single cells) the impact of genomic reads on expression levels is likely small. Notably, with increasing amounts of gDNA, the fraction of unmapped reads also increased (Additional file 1: Fig. S3B), suggesting that the presence of gDNA does decrease the quality of RNA-seq libraries and does influence which molecules are generated during cDNA generation.

We also analyzed the properties of the intronic reads in DNase-digested prime-seq libraries from HEK cells (Additional file 1: Fig. S4). Intronic reads are enriched towards the 3′ end of genes albeit not as strongly as exonic reads, suggesting that they are derived from internal as well as poly(A)-tail priming events (Additional file 1: Fig. S4). The probability of obtaining an intronic read from a gene depends probably on many factors, such as splicing dynamics (~10% of all transcripts are thought to be pre-mRNAs [46]), expression levels, efficiency of poly(A)-tail priming, and presence of internal priming sites. But as long as these reads are derived from RNA molecules, it seems reasonable to use them for quantifying and comparing gene expression levels as has been laid out previously [47].

In summary, these results indicate that essentially all reads in prime-seq libraries are derived from RNA when samples are DNase I treated and hence that intronic reads can be used to quantify expression levels.

### Prime-seq performs as well as TruSeq

Next, we quantitatively compared the performance of prime-seq to a standard bulk RNA-seq method with respect to library complexity, accuracy, and statistical power. A gold standard RNA-seq data set was generated in the third phase of the Microarray Quality Control (MAQC-III) study [48], consisting of deeply sequenced TruSeq RNA-seq libraries generated from five replicates of Universal Human Reference RNA (UHRR) and External RNA Controls Consortium (ERCC) spike-ins. As Illumina’s TruSeq protocol can be considered a standard bulk RNA-seq method, and as the reference RNAs (UHRR and ERCCs) are commercially available, this is an ideal data set to benchmark our method. As in the MAQC-III design, we mixed UHRR and ERCCs (Additional file 1: Fig. S5) in the same ratio but at a 1000-fold lower input and generated eight prime-seq libraries, which were sequenced to a depth of at least 30 million reads. We processed and downsampled both data using the zUMIs pipeline [45] and compared the two methods with respect to their library complexity (number and expression levels of detected genes), accuracy (correlation of estimated expression level and actual number of spiked-in ERCCs), and statistical power (true positive and false positive rates in data simulated based on the mean-variance distribution of technical replicates of each method).

**Fig. 3:**
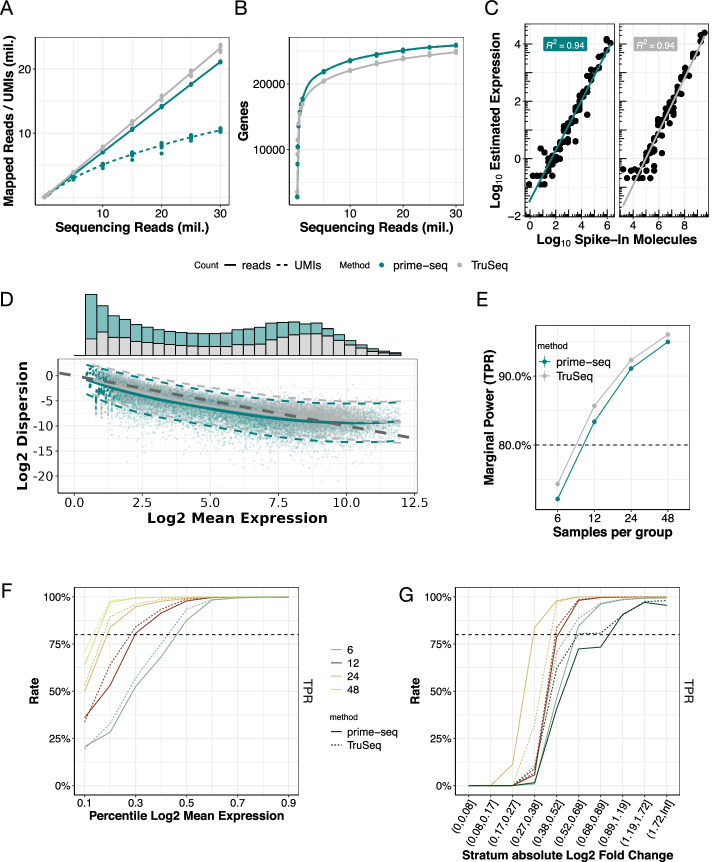
Prime-seq has similar sensitivity and power compared to TruSeq (MAQC-III data). **A** Mapped reads, UMIs (dashed line, only prime-seq), and **B** detected genes (exonic + intronic reads) at varying sequencing depths between TruSeq data from the MAQC-III Study and matched prime-seq data, show prime-seq and TruSeq are similarly sensitive (filtering parameters: detected UMI ≥ 1, detected gene present in at least 25% of samples and is protein coding). **C** Accuracy, measured by spike-in molecules, is similarly high in both methods (*R*^2^ = 0.94). **D** The distribution of genes across mean expression is similar for both methods, as well as the dispersion, which follows a Poisson distribution (dark gray dashed line) for lower expressed genes and then increases as technical variation increases for highly expressed genes. The local polynomial regression fit between mean and dispersion estimates per method is shown in solid lines with 95% variability band per gene shown in dashed lines. **E** Power analysis at a sequencing depth of 10 million reads shows almost identical power between prime-seq and TruSeq, and a similar increase at varying sample size for **F** mean expression and **G** absolute log2 fold change. Data filtering parameters: detected UMI ≥ 1, detected gene present in at least 25% of samples

We found that prime-seq has a slightly lower fraction of exonic and intronic reads that can be used to quantify gene expression (78% vs. 85%; Fig. 3A, Additional file 1: Fig. S6A). But despite the slightly lower number of reads that can be used, prime-seq does detect at least as many genes as TruSeq (Fig. 3B). Of these, 33,230 genes are detected with both methods (76.2%) (Additional file 1: Fig. S6B). Pairwise sample comparisons between (*R*^2^ = 0.64) the two methods are lower than within the methods (*R*^2^ = 0.94 and 0.97), as one would expect (Additional file 1: Fig. S6C). Additionally, the comparison of normalized expression data between prime-seq and TruSeq shows stronger correlation in ERCC spike-in molecules (*R*^2^ = 0.95) than endogenous molecules (*R*^2^ = 0.67) (Additional file 1: Fig. S6D). This is likely explained by the biological variation of the samples, as the ERCC spike-ins are synthetically produced to exact specifications, and UHRR is extracted from a mixture of cell lines, which may have altered in composition or expression in the 7 years separating the two experiments. Both methods also show a similar distribution of gene expression levels (Fig. 3D), indicating that the complexity of generated libraries is generally very similar.

The accuracy of a method, i.e., how well estimated expression levels reflect actual concentrations of mRNAs, is relevant when expression levels are compared among genes. Here, TruSeq and prime-seq show the same correlation (Pearson’s *R*^2^ = 0.94) between observed expression levels and the known concentration of ERCC spike-ins, indicating that their accuracy is very similar (Fig. 3C).

However, for most RNA-seq experiments, a comparison among samples—e.g., to detect differentially expressed genes—is more relevant. Therefore, it matters how well genes are measured by a particular method, i.e., how much technical variation a method generates across genes. As we have 8 and 5 technical replicates of the same RNA for prime-seq and TruSeq, respectively, we can estimate for each method the mean and variance per gene. Note that UMIs are only available for prime-seq and hence only prime-seq can profit from removing technical variance by removing PCR duplicates (Fig. 3A). The empirical distribution shows the characteristic dependency of RNA-seq data on sampling (Poisson expectation) at low expression levels and an increasing influence of the additional technical variation at higher expression levels (Fig. 3D). Prime-seq shows a slightly lower variance for medium expression levels where most genes are expressed (Fig. 3D). To quantify to what extent these differences in the mean-variance distribution actually matter, we used power simulations as implemented in powsimR [49]. We simulated that 10% of genes sampled from the estimated mean-variance relation of each method are differentially expressed between two groups of samples. The fold changes of these genes were drawn from a distribution similar to those we observed in actual data between two cell types (iPSCs and NPCs) or two types of acute myeloid leukemia (AML) (see below and Additional file 1: Fig. S7A). The comparison between this ground truth and the identified differentially expressed genes in a simulation allows us to estimate the true positive rate (TPR) and the false discovery rate (FDR) for a particular parameter setting. We stratified TPR and FDR across the number of replicates (Fig. 3E), the expression levels (Fig. 3F), and the fold changes (Fig. 3G) to illustrate the strong dependence of power on these parameters. At a given FDR level, a more powerful method reaches a TPR of 80% with fewer replicates, at a lower expression level, and/or for a lower fold change. We find that the power of the two methods is almost identical as FDR and TPR are very similar across conditions for both methods. The false discovery rates (FDR) are—as expected—generally below 5% for 12, 24, or 48 replicates per condition (Additional file 1: Fig. S7B-D) and the (marginal) TPR across all expression levels and fold changes is 80% for both methods at ~12 replicates per condition (Fig. 3E). The power increases for both methods in a similar manner with increasing expression levels (Fig. 3F) and increasing fold changes (Fig. 3G). This is also the case when using only exonic reads for the power analysis (Additional file 1: Fig. S7B and S7E-F). In summary, prime-seq and TruSeq perform very similarly in estimating gene expression levels with respect to library complexity, accuracy, and statistical power.

### Bead-based RNA extraction increases cost efficiency and throughput

**Fig. 4:**
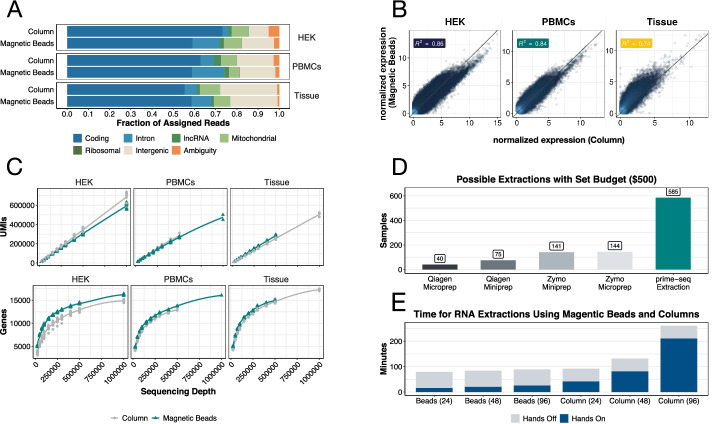
RNA extraction with beads, rather than columns, provides similar sequencing data while increasing throughput capabilities. **A** Feature distributions of RNA isolated with a column-based kit and magnetic beads show that both RNA extraction protocols produce similar amounts of useable reads from cultured human embryonic kidney 293T (HEK293T) cells, peripheral blood mononuclear cells (PBMC), and harvested mouse brain tissue. **B** Gene expression between both bead and column extraction are also similar in all three tested inputs (*R*^2^ = 0.86 HEK, 0.84 PBMCs, and 0.74 tissue). **C** Detected UMIs and detected genes for column and magnetic beads in HEK293T, PBMCs, and tissue are almost identical, with slightly more detected genes in the bead condition (filtering parameters: detected UMI ≥ 1, detected gene present in at least 25% of samples and is protein coding). Comparison of costs (**D**) and time (**E**) required for different RNA extractions

As library costs and sequencing costs drop, standard RNA isolation becomes a considerable factor for the cost efficiency of RNA-seq methods. RNA isolation using magnetic beads is an attractive alternative [50] and we have used it successfully in combination with our protocol before [11]. To investigate the effects of RNA extraction more systematically, we compared prime-seq libraries generated from RNA extracted via silica columns and via affordable carboxylated magnetic beads (for more information see Additional file 3. Supplemental Text). Libraries from cultured HEK293T cells, human peripheral blood mononuclear cells (PBMC), and mouse brain tissue showed a similar distribution of mapped reads, albeit with a slightly higher fraction of intronic reads in magnetic bead libraries (Fig. 4A and S8) and considerable differences in expression levels (Fig. 4B and S9).

To further explore these differences, we tested the influence of the Proteinase K digestion and its associated heat incubation (50 °C for 15 min and 75 °C for 10 min), which is part of the bead-based RNA isolation protocol. We prepared prime-seq libraries using HEK293T RNA extracted via silica columns (“Column”), magnetic beads with Proteinase K digestion (“Magnetic Beads”), magnetic beads without Proteinase K digestion (“No Incubation”), and magnetic beads with the same incubations but without the addition of the enzyme (“Incubation”). Interestingly, the shift to higher intronic fractions and the expression profile similarity is mainly due to the heat incubation, rather than the enzymatic digestion by Proteinase K (Additional file 1: Fig. S8A and B).

Hence, bead-based extraction does create a different expression profile than column-based extraction, especially due to the often necessary Proteinase K incubation step. This confirms the general influence of RNA extraction protocols on gene expression profiles [51]. Importantly, the complexity of the two types of libraries is similar, with a slightly higher number of genes detected in the bead-based isolation (Fig. 4C, Additional file 1: Fig. S8C and S8D), potentially due to a preference for longer transcripts with lower GC contents (Additional file 1: Fig. S9C).

So while bead-based RNA isolation and column-based RNA isolation create different but similarly complex expression profiles, bead-based RNA isolation has the advantage of being much more cost-efficient. At least four times more RNA samples can be processed for the same budget (Fig. 4D, Additional file 4: Table S2). In addition, RNA isolation using magnetic beads is twice as fast and without robotics more amenable to high-throughput experiments (Additional file 5: Table S3). Thus, we show that bead-based RNA isolation can make prime-seq considerably more cost-efficient without compromising library quality.

### Prime-seq is sensitive and works well with 1000 cells

As prime-seq was developed from a scRNA-seq method [44], it is very sensitive, i.e., it generates complex libraries from one or very few cells. This makes it useful when input material is limited, e.g., when working with rare cell types isolated by FACS or when working with patient material. To validate a range of input amounts, we generated RNA-seq libraries from 1000 (low input, ~10–20 ng total RNA) and 10,000 (high input, ~100–200 ng) HEK293T cells. The complexity of the two types of libraries was very similar, with only a 2% decrease in the fraction of exonic and intronic reads and a 7.7% and 1.9% reduction in the number of UMIs and detected genes at the same sequencing depth (Additional file 1: Fig. S10A). The expression profiles were almost as similar between the two input conditions as within the input conditions (median r within = 0.94, median *r* between = 0.93; Additional file 1: Fig. S10B), indicating that expression profiles from 1000 and 10,000 cells are almost identical in prime-seq. Using a lower number of input cells is certainly possible and unproblematic as long as the number of cells is unbiased with respect to the variable of interest. Using higher amounts than 10,000 cells is certainly also possible, but it is noteworthy that we have observed a large fraction of intergenic reads in highly concentrated samples, potentially due to incomplete DNase I digestion (data not shown). In summary, we validate that an input amount of at least 1000 cells does not compromise the complexity of prime-seq libraries and hence that prime-seq is a very sensitive RNA-seq protocol.

### Barcode swapping in prime-seq is low

One potential concern with early barcoding methods is the swapping of barcodes due to the formation of chimeric molecules during PCR, resulting in a “contamination” of a cell’s expression profile with transcripts from another cell. This has been discussed in the context of scRNA-seq library generation [52, 53], but it is not clear to what extent it is relevant in bulk RNA-seq methods. To quantify barcode swapping, we generated prime-seq libraries from isolated total RNA from mouse embryonic stem cells (mESCs) and human-induced pluripotent stem cells (iPSCs) either separately or pooled after reverse transcription (pooling) as it is normally done in the prime-seq protocol (Additional file 1: Fig. S11A). We find that less than 0.1% of the mapped UMIs in the ten separately amplified human libraries, map to mouse, representing a low background rate due to mismapping and index swapping during sequencing. In contrast, ~0.5% of the mapped UMIs in the five human libraries that were generated together with five mouse libraries map to mouse (Additional file 1: Fig. S11B). So barcode swapping does occur, but at a relatively low level, consistent with previous findings for single human and mouse cells for our related mcSCBR-seq method [11] (Additional file 1: Fig. S11C) and that the amount of swapped barcodes correlates strongly with the amount of transcripts in the pool (Additional file 1: Fig. S11D). Importantly, even 10% of barcode swapping has fairly little influence on power as shown in simulations (Additional file 1: Fig. S11E). In summary, we show that barcode swapping is present, but not a major issue for prime-seq as long as absolute expression levels, like the presence or absence of a gene, are interpreted accordingly. However, the amount of barcode swapping does depend on reaction conditions, specifically on the number of PCR cycles, but probably on more conditions such as types of polymerases [54], input amounts, library complexity, and sequence similarities. Hence, better controlling and understanding barcode swapping within and across methods might be important.

### Two exemplary applications of prime-seq

**Fig. 5:**
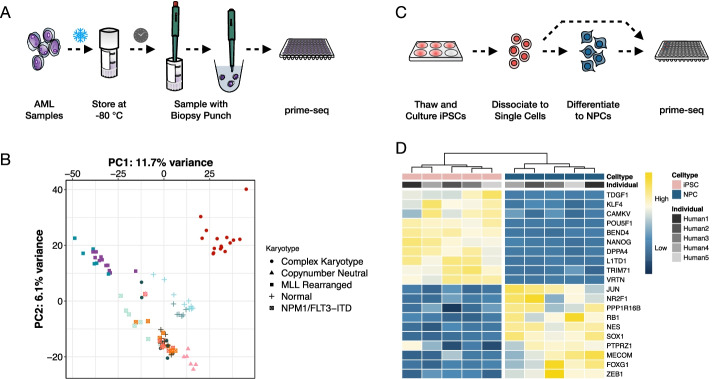
Two exemplary applications of prime-seq. **A** Experimental design for an acute myeloid leukemia (AML) study, where a biopsy punch was used to collect a small fraction of a frozen patient-derived xenograft (PDX)-AML sample. **B** Prime-seq libraries were generated from 94 PDX samples, derived from 11 different AML-PDX lines (color-coded) from 5 different AML subtypes (symbol-coded) and cluster primarily by AML subtype. **C** Experimental design for studying the differentiation from five human-induced pluripotent stem cell lines (iPSCs) to neural progenitor cells (NPC). **D** Expression levels from 20 a priori known marker genes cluster iPSCs and NPCs as expected

To exemplify the advantages with respect to sensitivity and throughput in an actual setting, we used prime-seq to profile cryopreserved human acute myeloid leukemia (AML) cells from patient-derived xenograft (PDX) models [23, 55]. These consisted of different donors and AML subtypes and were stored in freezing medium at – 80 °C for up to 3.5 years (Fig. 5A). Due to the sensitivity of prime-seq, we could use a minimal fraction of the sample without thawing it by taking a 1-mm biopsy punch from the vial of cryopreserved cells and putting it directly into the lysis buffer. This allowed sampling of precious samples without compromising their amount or quality and resulted in 94 high-quality expression profiles that clustered mainly by AML subtype (Fig. 5B) as expected [56].

To further exemplify the performance of prime-seq, we investigated its ability to detect known differences in a well-established differentiation system [57]. We differentiated five human-induced pluripotent stem cell (iPSCs) lines [36] to neural progenitor cells (NPCs) and generated expression profiles using prime-seq (Fig. 5C). In a hierarchical clustering of well-known marker genes [58], the iPSCs and NPCs formed two distinct groups and the expression patterns were in agreement with their cellular identity. For example, the iPSC markers POU5F1, NANOG, and KLF4 showed an increased expression in the iPSCs and NES, SOX1, and FOXG1 in NPCs (Fig. 5D).

### Prime-seq is cost-efficient

We have shown above that the power, accuracy, and library complexity is similar between prime-seq and TruSeq. The performance and robustness of the prime-seq protocol has been demonstrated by the two examples above as well as its many applications using this or previous versions of the protocol [9, 23–35, 42, 43, 59, 60]. In summary, one could argue that prime-seq performs as well as TruSeq for quantifying gene expression levels. Other methods that generate tagged cDNA libraries using early barcoding have also been developed [16, 22, 61–64]. This includes BRB-seq that uses poly(A) priming and DNA-Pol I for second-strand synthesis and also performs similarly to TruSeq [22]. Decode-seq also uses poly(A) priming and template switching like prime-seq, but adds sample-specific barcodes and UMIs at the 5′ end [16]. In a direct comparison, Decode-seq performed slightly better than BRB-seq and due to a more flexible sequencing layout [16]. While slight differences in power, accuracy, and/or library complexity might exist among these protocols, cross-laboratory benchmarking on exactly the same samples as recently done, e.g., for scRNA-seq methods [5] or small RNA-seq methods [65], are probably needed to quantify such differences reliably. For now, it is probably fair to say that RNA-seq methods like BRB-seq, prime-seq, TruSeq, Smart-Seq, or Decode-seq all perform fairly equal with respect to quantifying gene expression levels. Hence, at a fixed budget, the cost per sample will determine to a large extent how many samples can be analyzed and hence how much biological insight can be gained.

**Fig. 6:**
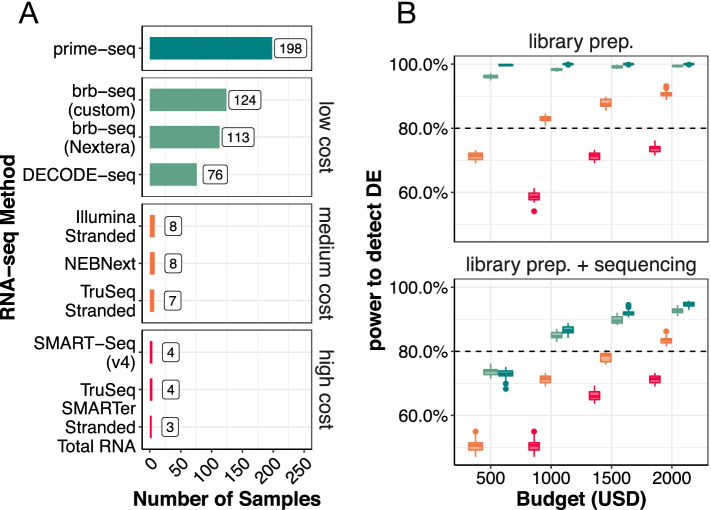
Prime-seq is very cost-efficient. **A** With a set budget of $500, prime-seq allows one to process 198 samples, which is 1.6 times more samples than the next cost-efficient method. **B** The compared methods were grouped into low, middle, and high cost methods and the TruSeq MAQCII data was used as a basis for power analysis for all methods but prime-seq. The increase in sample size due to cost efficiency directly impacts the power to detect differentially expressed genes, as evident by the increased performance of prime-seq and other low cost methods (BRB-seq and Decode-seq), even when sequencing costs are included in the comparison (sequencing depth of 10 mio. reads at a cost of $3.40 per 1 mio. reads)

To this end, we calculated the required reagent costs to generate a library from isolated RNA in a batch of 96 samples for the different commercial methods as well as for prime-seq, Decode-seq, and BRB-seq (Additional file 6 Table S4). With $2.53 per sample prime-seq is the most cost-efficient method, followed by BRB-seq ($4.05) and Decode-seq ($6.58). Commercial methods range from $60 (NEBNext) to $164 (SMARTer Stranded). This is illustrated by the number of libraries that can be generated by a fixed budget of $500 (Fig. 6A). Note that these costs include for all methods $1.39 per sample for two Bioanalyzer (Agilent) Chips (Additional file 6: Table S4) and do not consider the additional cost reduction that is associated with the direct bead-based RNA extraction of prime-seq (see above). The drastic advantage of prime-seq, Decode-seq, and BRB-seq also becomes apparent when power is plotted as a function of costs with and without sequencing (10 million reads per sample) (Fig. 6B, Additional file 1: Fig. S12A). For example, to reach an 80% TPR at a desired FDR of 5%, one needs to spend $715 including sequencing costs for prime-seq, $795 when using Decode-seq, $1625 when using Illumina Stranded, and $3485 when using TruSeq (Additional file 1: Fig. S12B).

Cost efficiency with respect to time can also matter and we calculated hands-on and hands-off time for the different methods (Additional file 7: Table S5). Hands-on times vary from 30 to 35 min for the non-commercial, early barcoding methods to 52–191 min for commercial methods. However, as all methods require essentially a full day of lab work, we consider the differences in required times not as decisive, at least not in a research lab setting where RNA-seq is not done on a daily or weekly basis. In summary, we find that prime-seq is the most cost-efficient bulk RNA-seq method currently available.

## Discussion

In this paper, we present and validate prime-seq, a bulk RNA-seq protocol, and show that it is as powerful and accurate as TruSeq in quantifying gene expression levels, but more sensitive and much more cost-efficient. We validate the DNase I treatment and determine that intronic reads are derived from RNA and can be used in downstream analysis. We also validate input ranges and the direct lysis and bead-based RNA purification of tissue and cell culture samples. Finally, we exemplify the use of prime-seq by profiling AML samples and NPC differentiation and show that prime-seq is currently the most cost-efficient bulk RNA-seq method. In the following, we focus our discussion on advantages and drawbacks of prime-seq in comparison to other RNA-seq protocols. To this end, we distinguish protocols like TruSeq, Smart-Seq, or NEBNext that individually process RNA samples and generate full-length cDNA profiles (“full-length protocols”) from protocols like prime-seq, Decode-seq, or BRB-seq that use early barcoding and generate 5′ or 3′ tagged cDNA libraries (“tag protocols”).

### Complexity, power, and accuracy are similar among most bulk RNA-seq protocols

Initially, early barcoding 3′ tagged protocols generated slightly less complex libraries (i.e., detected fewer genes for the same number of reads), especially due to a considerable fraction of unmapped reads [22, 66]. These reads are probably caused by PCR artifacts during cDNA generation and amplification. Protocol optimizations as shown for BRB-seq [22], Decode-seq [16], and here for prime-seq have reduced these artifacts and hence have improved library complexity to the level of standard full-length protocols. For prime-seq, we have shown quantitatively that its complexity, accuracy, and power is very similar to that of TruSeq. More comprehensive studies, ideally across laboratories [5, 48], would be needed to quantitatively compare protocols, also with respect to their robustness across laboratories and conditions and their biases for individual transcripts. For the context and methods discussed here, we would argue that there are no decisive differences in power, accuracy, and complexity among tag protocols and full-length protocols at least when performed under validated and optimized conditions.

### Cost efficiency makes tag-protocols preferable when quantifying gene expression levels

As shown above (Fig. 6) and as argued before [16, 22, 66], the main advantage of tag protocols is their cost efficiency. Their most obvious drawback is that they cannot quantify expression levels of different isoforms. Smart-Seq2 [67] and Smart-Seq3 [10] are relatively cost-efficient full-length protocols that were developed for scRNA-seq. However, they have not been validated and optimized for bulk RNA-seq and would still be considerably more expensive than most tag protocols. Furthermore, as reconstructing transcripts from short-read data is difficult and requires deep sequencing, isoform detection and quantification is now probably more efficiently done by using long-read technologies [1]. However, from our experience, most RNA-seq projects quantify expression at the gene level not at the transcript level. This is probably because most projects use RNA-seq to identify affected biological processes or pathways by a factor of interest. As different genes are associated with different biological processes, but different isoforms are only very rarely associated with different biological processes, most projects do not profit much from quantifying isoforms. Hence, we would argue that quantifying expression levels of genes is the better option, as long as isoform quantification is not of explicit relevance for a project.

Another limitation is that all tag-protocols use poly(A) priming and hence do not capture mRNA from bacteria, organelles, or other non-polyadenylated transcripts. For full-length protocols like TruSeq, cDNA generation by random priming after rRNA depletion can be done. Another possibility is poly(A) tailing after rRNA depletion [68], but to our knowledge, this has not been adopted to tag-based protocols yet. How to efficiently combine profiling of polyadenylated, non-polyadenylated, and small RNA is certainly worth further investigating. However, it is also true that for eukaryotic cells, quantification of mRNAs contains most of the information. Hence, similar to the quantification of isoforms, we would argue that quantifying expression levels of genes by polyadenylated transcript is often sufficient, as long as non-polyadenylated transcripts are not explicitly relevant.

Furthermore, early barcoding and pooling necessitates calibrating input amounts. Input calibration is easy when starting with extracted RNA or when it is possible to count cells prior to direct lysis. When counting cells is not possible, we have also developed a protocol adaptation of prime-seq that allows for RNA quantification and normalization after bead-based RNA isolation and prior to reverse transcription (10.17504/protocols.io.s9veh66).

Finally, early barcoding and pooling can lead to barcode swapping. We have shown that barcode swapping is not a major issue for prime-seq, but the amount of barcode swapping is unknown for most tag-protocols. However, even rather high levels of barcode swapping have a much smaller impact on power than a decrease in sample size (Additional file 1: Fig. S11E) and as long as the interpretation of absolute expression levels (e.g., presence/absence) is not crucial, the cost efficiency of tag-based protocols outweighs this drawback.

In summary, when quantification of isoforms and/or non-polyadenylated RNA is not necessary, a technically validated tag protocol has no drawbacks. Protocols that use poly(A) priming and template switching also have the advantage that they are very sensitive, and for prime-seq, we have validated that it still works optimally also with 1000 cells (~10–20 ng total RNA) as input. However, the decisive advantage of tag protocols is their drastically higher cost efficiency (Fig. 6), as this leads to drastically higher power and much more flexibility in the experimental design for a given budget. As repeated by biostatisticians over the decades, a good experimental design and a sufficient number of replicates is the most decisive factor for expression profiling. It is sobering how enduring the *n* = 3 tradition is, as is nicely shown in [16], although it is known that it is better to distribute the same number of reads across more biological replicates [17]. Cost-efficient tag protocols will hopefully make such experimental designs more common. While library costs are less notable for sequencing depths of 10 M reads or more (Fig. 6B), they may enable RNA-seq experiments that can be done with shallow sequencing, something which is less obvious and might be overlooked. Replacing qPCR has been advocated as one example by the authors of BRB-seq [22]. But also other applications, like characterizing cell type composition [36], quality control of libraries, or optimizing experimental procedures can profit considerably from low library costs.

In summary, tag protocols allow flexible designs of RNA-seq experiments that should be helpful for many biological questions and have a vast potential when readily accessible for many labs.

### Validation, documentation, and cost efficiency make prime-seq a good option for setting up a tag protocol

We have argued above that adding a tag protocol to the standard method repertoire of a molecular biology lab is advantageous due to its cost efficiency. As the different tag protocols discussed here perform fairly similar with respect to complexity, power, accuracy, sensitivity, and cost efficiency, essentially any of them would suffice. If one has a validated, robust protocol running in a lab or core facility, it is probably not worth switching. That said, our results might still help to better validate existing protocols, integrate direct lysis, and make use of intronic reads. If one does not have a tag protocol running, we would argue that our results provide helpful information to decide on a protocol and that prime-seq would be a good option for several reasons as laid out in the following.

A main difference among tag protocols is whether they tag the 5′ end, like Decode-seq, or tag the 3′ end like BRB-seq or prime-seq. 5′ tagging has some obvious advantages (see also [16]), including the possibility to read both ends of the cDNA as one cannot read through the poly(A) tail. Using the sequence information from the 5′ end is also important to distinguish alleles of B-cell receptors and T-cell receptors [69]. In scRNA-seq, both 5′ and 3′ tag protocols have been successfully used, but 3′ tagging is currently the standard. The reason for this is not obvious, but it might be that the incorporation of the barcode and the UMI is more difficult to optimize [10]. Additionally, the higher level of alternative splicing at the 5′ end could make gene-level quantification more difficult. More dedicated comparisons would be needed to further investigate these factors. Currently, 3′ tag protocols are more established and when using a suitable sequencing design, poly(A) priming does not compromise sequencing quality as validated by us and the widespread use of Chromium 10x v3 chemistry scRNA-seq libraries that have the same layout as prime-seq.

As shown above, prime-seq is among all protocols the most cost-efficient when starting from purified RNA. It is also currently the only protocol for which a direct lysis is validated, which further increases cost efficiency of library production. This is especially advantageous when processing many samples, shallow sequencing is sufficient, and/or as sequencing costs continue to drop.

Finally, we think that prime-seq is the easiest tag protocol to set up. While many such protocols have been published and all have argued that their method would be useful, few have actually become widely implemented. The reasons are in all likelihood complex, but we think that prime-seq has the lowest barriers to be set up by an individual lab or a core facility for three reasons: First, to our knowledge, it is the most validated non-commercial bulk RNA-seq protocol, based on the experiments presented here as well as our >5 years of experience in running various versions of the protocol with over 6000 samples across 17 species resulting in over 20 publications to date. It is the only protocol for which direct lysis and sensitivity are quantitatively validated. Also, it is well validated in combination with zUMIs, the computational pipeline that was developed and is maintained by our group [45]. Second, it is not only cost-efficient per sample, but it also has low setup costs. It requires no specialized equipment and only the barcoded primers as an initial investment of ~$2000 for 96 primers, which will be sufficient for processing more than 240,000 samples. Finally, prime-seq is well documented not only by this manuscript, but also by a step-by-step protocol, including all materials, expected results, and alternative versions depending on the type and amounts of input material (10.17504/protocols.io.s9veh66). Hence, we think that prime-seq is not only a very useful protocol in principle, but also in practice.

## Conclusion

The multi-dimensional phenotype of gene expression is highly informative for many biological and medical questions. As sequencing costs dropped, RNA-seq became a standard tool in investigating these questions. We argue that the decisive next step is to use the possibilities of lowered library costs by tag protocols to leverage even more of this potential. We show that prime-seq is currently the best option when establishing such a protocol as it performs as well as other established RNA-seq protocols with respect to its accuracy, power, and library complexity. Additionally, it is very sensitive, is well documented, and is the most cost-efficient bulk RNA-seq protocol currently available to set up and to run.

## Methods

A step-by-step protocol of prime-seq, including all materials and expected results, is available on protocols.io (10.17504/protocols.io.s9veh66). Below, we briefly outline the prime-seq protocol, as well as describe any experiment-specific methods and modifications that were made to prime-seq during testing and optimization.

### Prime-seq

Cell lysates, generally containing around 1000–10,000 cells, were treated with 20 μg of Proteinase K (Thermo Fisher, #AM2546) and 1 μL 25 mM EDTA (Thermo Fisher, EN0525) at 50 °C for 15 min with a heat inactivation step at 75 °C for 10 min. The samples were then cleaned using cleanup beads, a custom-made mixture containing SpeedBeads (GE65152105050250, Sigma-Aldrich), at a 1:2 ratio of lysate to beads. DNA was digested on-beads using 1 unit of DNase I (Thermo Fisher, EN0525) at 20 °C for 10 min with a heat inactivation step at 65 °C for 5 min.

The samples were then cleaned and the RNA was eluted with the 10 μL reverse transcription mix, consisting of 30 units Maxima H- enzyme (Thermo Fisher, EP0753), 1×  Maxima H- Buffer (Thermo Fisher), 1 mM each dNTPs (Thermo Fisher), 1 μM template-switching oligo (IDT), and 1 μM barcoded oligo (dT) primers (IDT). The reaction was incubated at 42 °C for 90 min.

Following cDNA synthesis, the samples were pooled, cleaned, and concentrated with cleanup beads at a 1:1 ratio and eluted in 17 μL of ddH_2_O. Residual primers were digested using Exonuclease I (Thermo Fisher, EN0581) at 37 °C for 20 min followed by a heat inactivation step at 80 °C for 10 min. The samples were cleaned once more using cleanup beads at a 1:1 ratio, and eluted in 20 μL of ddH_2_O.

Second-strand synthesis and pre-amplification were performed in a 50 μL reaction, consisting of 1× KAPA HiFi Ready Mix (Roche, 7958935001) and 0.6 μM SingV6 primer (IDT), with the following PCR setup: initial denaturation at 98 °C for 3 min, denaturation at 98 °C for 15 s, annealing at 65 °C for 30 s, elongation at 68 °C for 4 min, and a final elongation at 72 °C for 10 min. Denaturation, annealing, and elongation were repeated for 5–15 cycles depending on the initial input.

The DNA was cleaned using cleanup beads at a ratio of 1:0.8 of DNA to beads and eluted with 10 μL of ddH_2_O. The quantity was assessed using a Quant-iT PicoGreen dsDNA assay kit (Thermo Fisher, P11496) and the quality was assessed using an Agilent 2100 Bioanalyzer with a High-Sensitivity DNA analysis kit (Agilent, 5067-4626).

Libraries were prepared with the NEBNext Ultra II FS Library Preparation Kit (NEB, E6177S) according to the manufacturer instructions in most steps, with the exception of adapter sequence and reaction volumes. Fragmentation was performed on 2.5 μL of cDNA (generally 2–20 ng) using Enzyme Mix and Reaction buffer in a 6 μL reaction. A custom prime-seq adapter (1.5 μM, IDT) was ligated using the Ligation Master Mix and Ligation Enhancer in a reaction volume of 12.7 μL. The samples were then double-size selected using SPRI-select Beads (Beckman Coulter, B23317), with a high cutoff of 0.5 and a low cutoff of 0.7. The samples were then amplified using Q5 Master Mix (NEB, M0544L), 1 μL i7 Index primer (Sigma-Aldrich), and 1 μL i5 Index primer (IDT) using the following setup: 98 °C for 30 s; 10–12 cycles of 98 °C for 10 s, 65 °C for 1 min 15 s, 65 °C for 5 min; and 65 °C for 4 min. Double-size selection was performed once more as before using SPRI-select Beads. The quantity and quality of the libraries were assessed as before.

### Nextera XT Library Prep

Prior to using the NEBNext Ultra II FS Library Kit, libraries were prepared using the Nextera XT Kit (Illumina, FC-131-1096). This included the RNA extraction experiments (Fig. 4) as well as the AML experiment (Fig. 5B). These libraries were prepared as previously described [11].

Briefly, three replicates of 0.8 ng of DNA were tagmented in 20 μL reactions. Following tagmentation, the libraries were amplified using 0.1 μM P5NextPT5 primer (IDT) and 0.1 μM i7 index primer (IDT) in a reaction volume of 50 μL. The index PCR was incubated as follows: gap fill at 72 °C for 3 min, initial denaturation at 95 °C for 30 s, denaturation at 95 °C for 10 s, annealing at 62 °C for 30 s, elongation at 72 °C for 1 min, and a final elongation at 72 °C for 5 min. Denaturation, annealing, and elongation were repeated for 13 cycles.

Size selection was performed using gel electrophoresis. Libraries were loaded onto a 2% Agarose E-Gel EX (Invitrogen, G401002) and were excised between 300 and 900 bp and cleaned using the Monarch DNA Gel Extraction Kit (NEB, T1020). The libraries were quantified and qualified using an Agilent 2100 Bioanalyzer with a High-Sensitivity DNA analysis kit (Agilent, 5067-4626).

### Barcoded oligo (dT) primer design

In order to enable more robust demultiplexing and to ensure full compatibility of our sequencing layout with the Chromium 10x v3 chemistry, oligo (dT) primers were designed to include a 12 nt cell barcode and 16 nt UMI. Candidate cell barcodes were created in R using the DNABarcodes package [70] to generate barcodes with a length of 12 nucleotides and a minimum Hamming distance (HD) of 4, with filtering for self-complementarity, homo-triplets, and GC-balance enabled. Candidate barcodes were filtered further, resulting in a barcode pool with a minimal HD of 5 and a minimal Sequence-Levenshtein distance of 4 within the set. In order to balance nucleotide compositions among cell barcodes at each position, BARCOSEL [71] was used to further reduce the candidate set down to the final 384 barcodes.

### Sequencing

Sequencing was performed on an Illumina HiSeq 1500 instrument for all libraries except for the IPSC/NPC experiment where a NextSeq 550 instrument was used. The following setup was used: Read 1: 28 bp, Index 1: 8 bp; Read 2: 50-56 bp.

### Pre-processing of RNA-seq data

The raw data was quality checked using fastqc (version 0.11.8 [72]) and then trimmed of poly(A) tails using Cutadapt (version 1.12, 10.14806/ej.17.1.200). Following trimming, the zUMIs pipeline (version 2.9.4 ,[45]) was used to filter the data, with a Phred quality score threshold of 20 for 2 BC bases and 3 UMI bases. The filtered data was mapped to the human genome (GRCh38) with the Gencode annotation (v35) or the mouse genome (GRCm38) with the Gencode annotation (vM25) using STAR (version 2.7.3a,[73]) and the reads counted using RSubread (version 1.32.4,[74]).

### Sensitivity and differential gene expression analysis of RNA-seq data

The count matrix generated by zUMIs was loaded into RStudio (version 1.3.1093 [75]) using R (version 4.0.3 [76]). bioMart (version 2.46.0 [77]), dplyr (version 1.0.2 [78]), and tidyr (version 1.1.2 [79]) were used for data processing and calculating descriptive statistics (i.e., detected genes, reads, and UMIs). DESeq2 (version 1.30.0 [80]) was used for differential gene expression analysis. ggplot2 (version 3.3.3 [81]), cowplot (version 1.1.1 [82]), ggbeeswarm (0.6.0 [83]), ggsignif (version 0.6.0 [84]), ggsci (version 2.9 [85]), ggrepel (version 0.9.0 [86]), EnhancedVolcano (1.8.0 [87]), ggpointdensity (version 0.1.0 [88]), and pheatmap (version 1.0.12 [89]) were used for data visualization.

### Power analysis of RNA-seq data

Power simulations were performed following the workflow of the powsimR package (version 1.2.3 [49]). Briefly, RNA-seq data per method was simulated based on parameters extracted from the UHRR comparison experiment. For each method and sample size setup (6 vs. 6, 12 vs. 12, 24 vs. 24, and 48 vs. 48), 20 simulations were performed with the following settings: normalization = “MR,” RNA-seq = “bulk,” Protocol = “Read/UMI,” Distribution = “NB,” ngenes = 30000, nsims = 20, p.DE = 0.10. We verified with the data generated from the AML and NPC differentiation data that the gamma distribution (shape = 1, scale = 0.5) would be an appropriate log fold change distribution in this case (Additional file 1: Fig. S7A).

To simulate contamination by cross-contamination, we assumed that contamination increases with expression as shown in Additional file 1: Fig. S11D and can thus be simulated by sampling from the overall counts per gene in a pool. Different levels of contamination (0.5%, 1%, 2.5%, 5%, 10%) were simulated and added to the original count matrix. Power simulations were run as described above.

### Cell preparation

Human embryonic kidney 293T (HEK293T) cells were cultured in DMEM media (TH.Geyer, L0102) supplemented with 10% FBS (Thermo Fisher, 10500-064) and 100 U/ml Penicillin and 100 μg/ml Streptomycin (Thermo Fisher). Cells were grown to 80% confluency and harvested by trypsinization (Thermo Fisher, 25200072).

Peripheral blood mononuclear cells (PBMCs) were obtained from LGC Standards (PCS-800-011). Before use, the cells were thawed in a water bath at 37 °C and washed twice with PBS (Sigma-Aldrich, D8537).

Prior to lysis, cells were stained with 1 μg/ml Trypan Blue (Thermo Fisher Scientific, 15-250-061) and counted using a Neubauer counting chamber. Then, the desired number of cells (1000 or 10,000) was pelleted for 5 min at 200 rcf, resuspended in 50 μL of lysis buffer (RLT Plus (Qiagen, 1053393) and 1% ß-mercaptoethanol (Sigma-Aldrich,M3148) and transferred to a 96-well plate. Samples were then stored at − 80 °C until needed.

### Tissue preparation

Striatal tissue from C57BL/6 mice between the ages of 6 and 12 months was harvested by first placing the mouse in a container with Isoflurane (Abbot, TU 061220) until the mouse was visibly still and exhibited labored breathing. The mice were then removed from the container, and a cervical dislocation was performed. The mice were briefly washed with 80% EtOH, the head decapitated, and the brain removed. The brain was transferred to a dish with ice-cold PBS and placed in a 1-mm slicing matrix.

Using steel blades (Wilkinson Sword, 19/03/2016DA), 5 coronal incisions were made. Biopsy punches (Kai Medical, BPP-20F) were then taken from the striatum and the tissue was transferred to a 1.5-mL tube with 50 μL of lysis buffer, RLT Plus, and 1% ß-mercaptoethanol. The tubes were snap frozen and stored at − 80 °C until needed.

### RNA extraction experiments

To determine differences due to RNA extraction, we isolated RNA using columns from the Direct-zol RNA MicroPrep Kit (Zymo, R2062) (condition: “Column”) and magnetic beads from the prime-seq protocol (conditions: “No Incubation,” “Incubation,” and “Magnetic Beads”) (see above for details on prime-seq). For the “Column” condition, the manufacturer instructions were followed and both the Proteinase K and DNase digestion steps were performed as outlined in the protocol. For the magnetic bead isolation, the prime-seq protocol was used as outlined in the “Magnetic Beads” condition. For “No Incubation” condition, the Proteinase K digestion was skipped entirely. For the “Incubation” condition, the Proteinase K digestion was performed but with no enzyme; that is the heat cycling of 50 °C for 15 min and 75 °C for 10 min was carried out but no enzyme was added to the lysate.

### gDNA priming experiment

For a graphical overview of the gDNA Priming experiment, see Fig. 2B. Frozen vials of mouse embryonic stem cells (mESC), which have been cultured as previously described (citation Bagnoli) (clone J1, frozen in Bambanker (NIPPON Genetics, BB01) on 04.2017), and HEK293T cells (frozen in Bambanker on 30.11.18, passage 25) were thawed. DNA was extracted from 1 million mESCs using DNeasy Blood & Tissue Kit (Qiagen, 69506) and RNA was extracted from 450,000 HEK293T cells using the Direct-zol RNA MicroPrep Kit (Zymo, R2062), according to the manufacturer instructions in both cases. The optional DNase treatment step during the RNA extraction was performed in order to remove any residual DNA.

After isolating DNA and RNA, the two were mixed to obtain the following conditions: 10 ng RNA/ 7 ng DNA, 7.5 ng RNA/ 1.75 ng DNA, and 10 ng RNA/ 0 ng DNA. The 10 ng RNA/ 7 ng DNA condition, which represents the highest contamination of DNA, was performed twice, once without DNase treatment and once with DNase treatment. Libraries were prepared from three replicates for each condition using prime-seq and were then sequenced (see above for detailed information).

### MAQC-III comparison experiment

For a graphical overview of the experimental design, see Additional file 1: Fig. S5. As only Mix A from the original MAQC-III Study was compared, 122.2 μL of ddH_2_O, 2.8 μL of UHRR (100 ng/μL) (Thermo Fisher, QS0639), and 2.5 μL of ERCC Mix 1 (1:1000) (Thermo Fisher, 4456740) were combined to generate a 1:500 dilution of Mix A. Eight RNA-seq libraries were constructed using prime-seq (see above methods) with 5 μL of the 1:500 Mix A.

The samples were sequenced and the data processed and analyzed as outlined above. Of the comparison data from the original MAQC-III Study, Experiment SRX302130 to SRX302209 from Submission SRA090948 were used as this was the sequence data from one site (BGI) and was sequenced using an Illumina HiSeq 2000 [48]. The TruSeq data was first trimmed to be 50 bp long and then processed with zUMIs as outlined above, with the exception of using both cDNA reads and not providing UMIs as there were none. Paired-end data was used to not penalize TruSeq, as this is a feature of the method.

### Barcode swapping experiments

In order to estimate cross-contamination levels in prime-seq introduced by barcode swapping, we isolated RNA from human-induced pluripotent stem cells (line 29B5, passage 34) [60] and mouse ES cells (line JM8, passage 27) [2] using the Direct-zol RNA MicroPrep Kit (Zymo, R2062). RNA concentrations were measured using the QuantiFlour RNA Dye (Promega, E3310) and 8 ng of total RNA were added per well. For the experiment estimating the impact of amplification on contamination, different nanograms of RNA per well (0.5, 2, 8, 32, 128) were amplified with different numbers of cycles (17, 15, 13, 11, 9). Prime-seq was performed as described before with pooling of samples from the different species (Additional file 1: Fig. S11A). Contamination was assessed by mapping to a concatenated human and mouse genome and assigning reads to species based on which genome they mapped to best.

### NPC differentiation experiment

To differentiate hiPSCs to NPCs, cells were dissociated and 9 × 10^3^ cells were plated into each well of a low attachment U-bottom 96-well-plate in 8GMK medium consisting of GMEM (Thermo Fisher), 8% KSR (Thermo Fisher), 5.5 ml 100× NEAA (Thermo Fisher), 100 mM sodium pyruvate (Thermo Fisher), 50 mM 2-Mercaptoethanol (Thermo Fisher) supplemented with 500 nM A-83-01 (Sigma-Aldrich), 100 nM LDN 193189 (Sigma-Aldrich), and 30 μM Y27632 (biozol). A half-medium change was performed on days 2 and 4. On day 6, Neurospheres from 3 columns were pooled, dissociated using Accumax (Sigma-Aldrich) and seeded on Geltrex (Thermo Fisher) coated wells. After 2 days, cells were dissociated and counted and 2 × 10^4^ were lysed in 100 μL of lysis buffer (RLT Plus (Qiagen, 1053393) and 1% ß-mercaptoethanol (Sigma-Aldrich,M3148).

### AML-PDX sample collection

Acute myeloid leukemia (AML) cells were engrafted in NSG mice (The Jackson Laboratory, Bar Harbour, ME, USA) to establish patient-derived xenograft (PDX) cells [55]. AML-PDX cells were cryopreserved as 10 Mio cells in 1 mL of freezing medium (90% FBS, 10% DMSO) and stored at – 80 °C for biobanking purposes. To avoid thawing these samples and thus harming or even destroying them, the frozen cell stocks were first transferred to dry ice under a cell culture hood. Next a sterile 1-mm biopsy punch was used to punch the frozen cells in the vial and transfer the extracted cells to one well of a 96-well plate containing 100 μL RLTplus lysis buffer with 1% beta mercaptoethanol. To ensure complete lysis, the lysate was mixed and snap frozen on dry ice. One biopsy punch is estimated to contain 10 μL of cryopreserved cells corresponding to roughly 1 × 10^5 cells given an even distribution of cells within the original vial. All 96 samples were collected in this manner, biopsy punches were washed using RNAse Away (Thermo Fisher Scientific) and 80% Ethanol for reuse. These lysates were subjected to prime-seq, including RNA isolation using SPRI beads. In total, PDX samples from 11 different AML patients were analyzed in 6 to 16 biological replicates (engrafted mice) per sample.

### Cost comparisons

Costs were determined by searching for general list prices from various vendors. When step by step protocols were available, each component was included in the cost calculation, such as for the SMARTer Stranded Total RNA Kit (Takara, 634862), SMART-Seq RNA Kit (v4) (Takara, 634891), TruSeq Library Prep (Illumina, RS-122-2001/2), TruSeq Stranded Library Prep (Illumina, 20020595), and Illumina Stranded mRNA Prep (Illumina, 20040534). In the case of BRB-seq, no publicly available step-by-step protocol was found, so the methods section was used to calculate costs [22]. Decode-seq has a publicly available protocol; however, the level of detail was insufficient to calculate exact costs; therefore, when specific vendors were not listed, we used the most affordable option that we have previously validated. In all cases, the prices included sales tax and were listed in euros and were therefore converted to USD using a conversion rate of 1.23 USD to EUR. The costs for all methods can be found in Table S4.

## Supplementary Information

**Additional file 1: Fig. S1**. Molecular workflow of prime-seq. **Fig. S2**. prime-seq is a robust protocol and has been validated with numerous organisms. **Fig. S3**. Intronic reads are not derived from contaminating gDNA. **Fig. S4**. Intron counts are enriched at the 3’ prime end and correlate with exon counts. **Fig. S5**. Experimental design comparing prime-seq to TruSeq data generated in the MAQC-III Study. **Fig. S6**. prime-seq and TruSeq have similar mapping, gene detection, and expression. **Fig. S7**. Power and FDR mostly depend on sample size and are similar between prime-seq and TruSeq. **Fig. S8**. Performance of isolation methods is similar independent of prefiltering or usage of only Exon data. **Fig. S9**. Most genes are detected independent of the extraction method used. **Fig. S10**. prime-seq performs equally well with high- and low-input samples. **Fig. S11**. Cross-contamination levels are low, increase with additional cycles but do not impact power simulations. **Fig. S12**. Power analysis shows prime-seq is able to reach 80% power earlier than less cost-efficient methods.

**Additional file 2: Table S1**. (Sensitivity) List of experiments performed with prime-seq including key characteristics of the experiments and data quality.

**Additional file 3: Supplemental Text**. Magnetic Beads used in prime-seq.

**Additional file 4: Table S2**. (Lysis Costs) Calculations for per sample costs of different commercial and non-commercial extraction methods.

**Additional file 5: Table S3**. (Lysis Time) Time needed for extraction of 24, 48 and 96 samples with SPRI beads or Silica Columns.

**Additional file 6: Table S4**. (Method Cost) Per sample cost calculations for popular commercial and non-commercial RNA-seq methods including all consumables and reagents.

**Additional File 7: Table S5**. (Method Time) Time needed for performing for popular commercial and non-commercial RNA-seq methods.

**Additional file 8.** Review history.

## Data Availability

The datasets generated and/or analyzed during the current study are available in the ArrayExpress repository under the following accession numbers E-MTAB-10133, 10138-10142, 10175, 11455, 11456 [90–98]. The MAQC-III Study, Experiment SRX302130 to SRX302209 from Submission SRA090948 were retrieved from the short-read archive [99]. The code required to generate the figures can be found at https://github.com/Hellmann-Lab/prime-seq [100] (published under GPL-3 License). A stable version of the github repository is available through zenodo (10.5281/zenodo.5932624) [101].
