# Supplementary material for: Prime-seq, efficient and powerful bulk RNA sequencing
Source: Genome Biol. 2022 Mar 31;23:88. doi: 10.1186/s13059-022-02660-8 (PMC8969310; doi:10.1186/s13059-022-02660-8)
Supplement: Supplementary file 1 — Additional file 1: Fig. S1. Molecular workflow of prime-seq. Fig. S2. prime-seq is a robust protocol and has been validated with numerous organisms. Fig. S3. Intronic reads are not derived from contaminating gDNA. Fig. S4. Intron counts are enriched at the 3’ prime end and correlate with exon counts. Fig. S5. Experimental design comparing prime-seq to TruSeq data generated in the MAQC-III Study. Fig. S6. prime-seq and TruSeq have similar mapping, gene detection, and expression. Fig. S7. Power and FDR mostly depend on sample size and are similar between prime-seq and TruSeq. Fig. S8. Performance of isolation methods is similar independent of prefiltering or usage of only Exon data. Fig. S9. Most genes are detected independent of the extraction method used. Fig. S10. prime-seq performs equally well with high- and low-input samples. Fig. S11. Cross-contamination levels are low, increase with additional cycles but do not impact power simulations. Fig. S12. Power analysis shows prime-seq is able to reach 80% power earlier than less cost-efficient methods. [file 13059_2022_2660_MOESM1_ESM.docx]

**
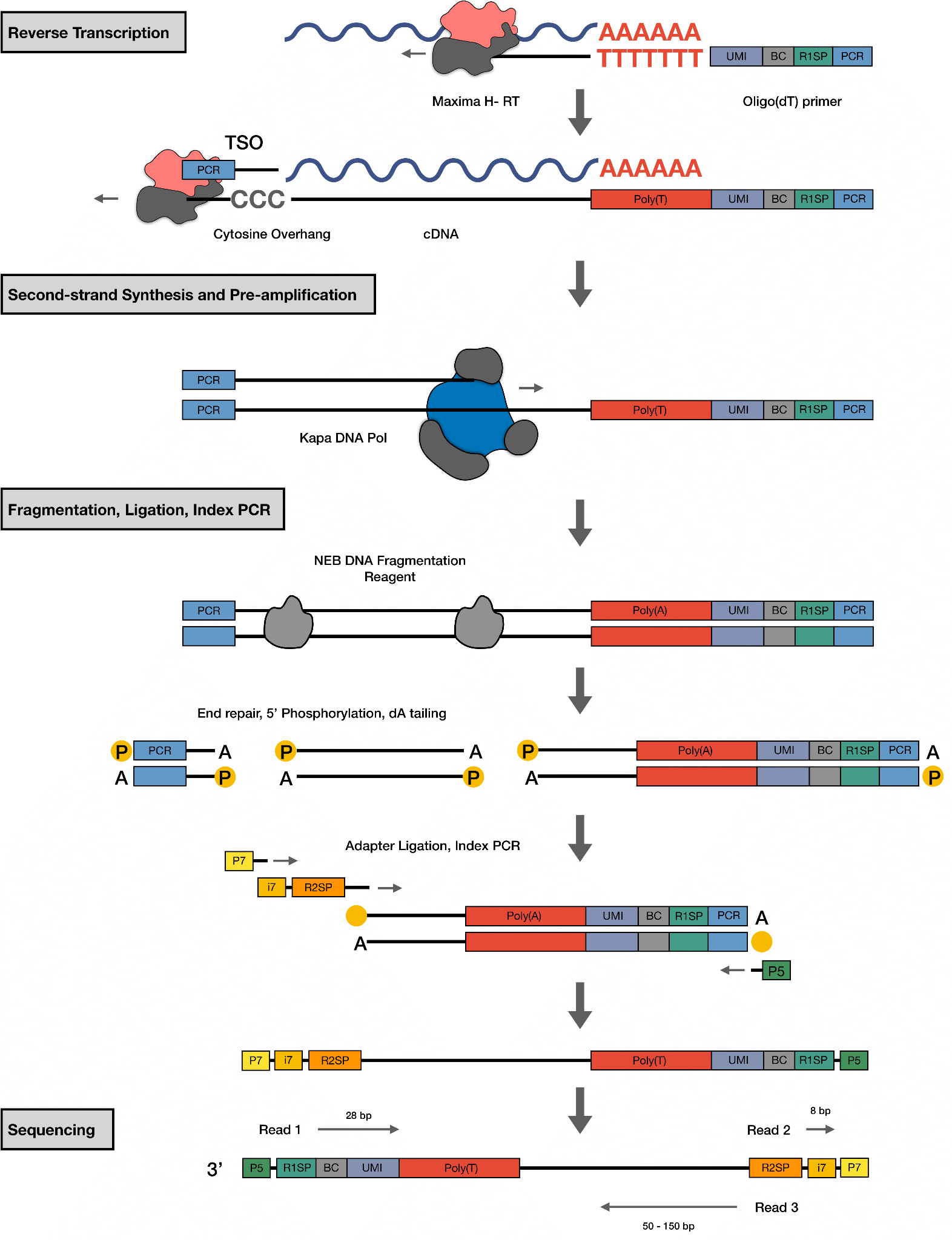
**

**Fig. S1. Molecular workflow of prime-seq.** (Related to Figure 1) oligo(dT)-primers are used to enrich mRNA, which is then reverse transcribed using Maxima H-, a M-MLV reverse transcriptase. Full length first strand synthesis is performed using a template switching oligo. Second strand synthesis and cDNA pre-amplification is completed during the PCR using KAPA Hifi Polymerase, and this DNA is then used to generate libraries using the NEBNEXT Ultra II FS Kit. Finally the libraries are sequenced with the following setup: read 1: 28bp, read 2: 8bp, and read 3: 50-150bp.

**
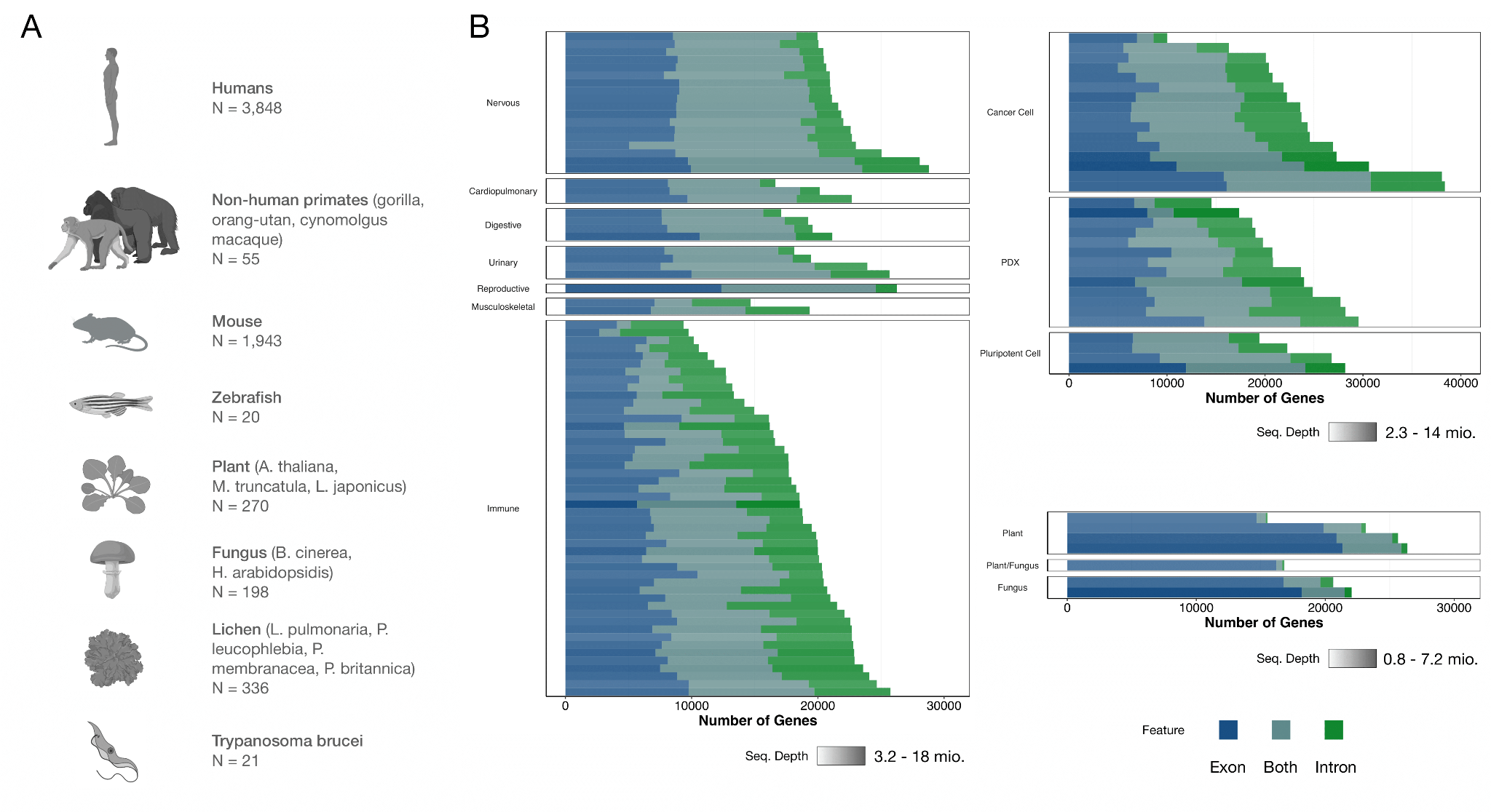
**

**Fig. S2. prime-seq is a robust protocol and has been validated with numerous organisms.** (Related to Figure 2A) (A) To date, 132 experiments consisting of 6,691 samples from 17 different organisms, ranging from arabidopsis to zebrafish, have been processed with prime-seq. (B) Data from experiments with well-annotated genomes suggests a substantial number of detected genes come from intronic reads.


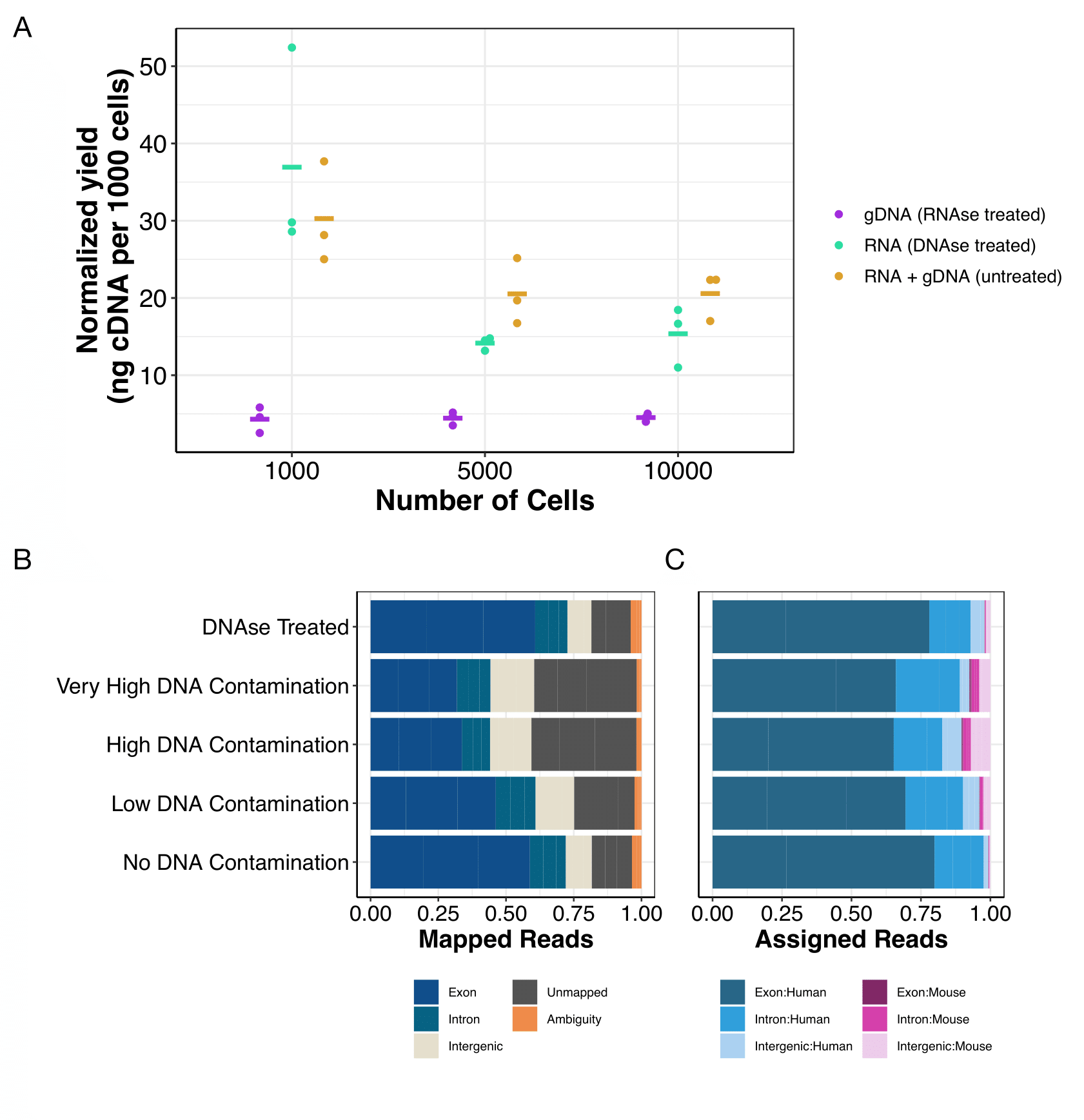


**Fig. S3. Intronic reads are not derived from contaminating gDNA.** (A) Samples containing total nucleic acids were either treated with RNase A or DNase I, or remained untreated. Untreated samples had the highest concentration, showing that genomic DNA is also used as a template when not removed, albeit less efficiently than mRNA. cDNA yields were normalized to the number of input cells. (Related to Figure 2B) (B) Mapped reads from different gDNA/RNA mixed conditions, showing that the DNase treated condition and the no DNA contamination condition had the lowest fraction of intergenic and unmapped reads. (C) Fraction of assigned mapped reads per genomic feature (exon, intron, intergenic) and species, showing an increase in mouse reads with higher gDNA contamination.

**
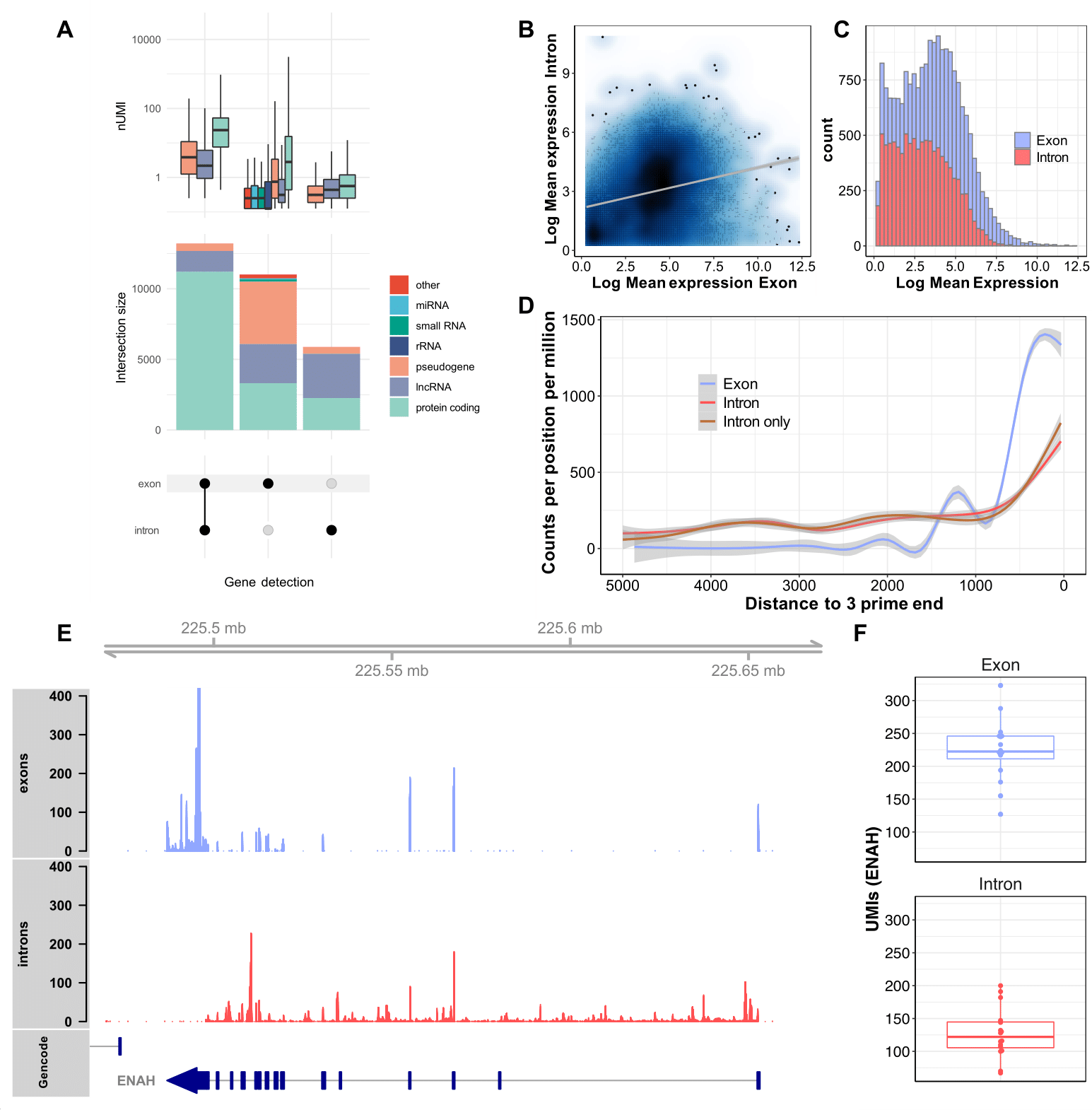
**

**Fig. S4. Intron counts are enriched at the 3' prime end and correlate with exon counts.** (A) Upset plot showing the intersection of genes detected with reads mapped to exons, introns or both. Most genes are detected in both introns and exons, followed by exons and introns only. Color represents the biotypes of the detected genes. Genes detected in both introns and exons are enriched for protein coding genes. Boxplots above show the expression levels of the genes by biotype. Genes detected with both intron and exon mapped reads are most highly expressed, intron only detected genes are lowly expressed. (B) Mean expression based on exon counts shows weak correlation to intron counts. (C) Histograms of expression levels of exon counts and intron counts normalized to total counts (intron plus exon) show higher average expression for exon counts. (D) 3’ prime enrichment of exon counts, intron counts and intron only counts. Counts per position relative to the 3’ prime per million averaged over 2000 genes with highest overall expression. Exon and intron counts are enriched at the 3’ prime end of the genbody. Intron only counts follow the same pattern as intron counts in genes with exon counts. (E) Exemplary exon and intron coverage for the gene ENAH show mapping of the intron counts coincides with mapping of exon counts along the gene body. (F) Corresponding UMI counts of ENAH based on intron and exon counting.


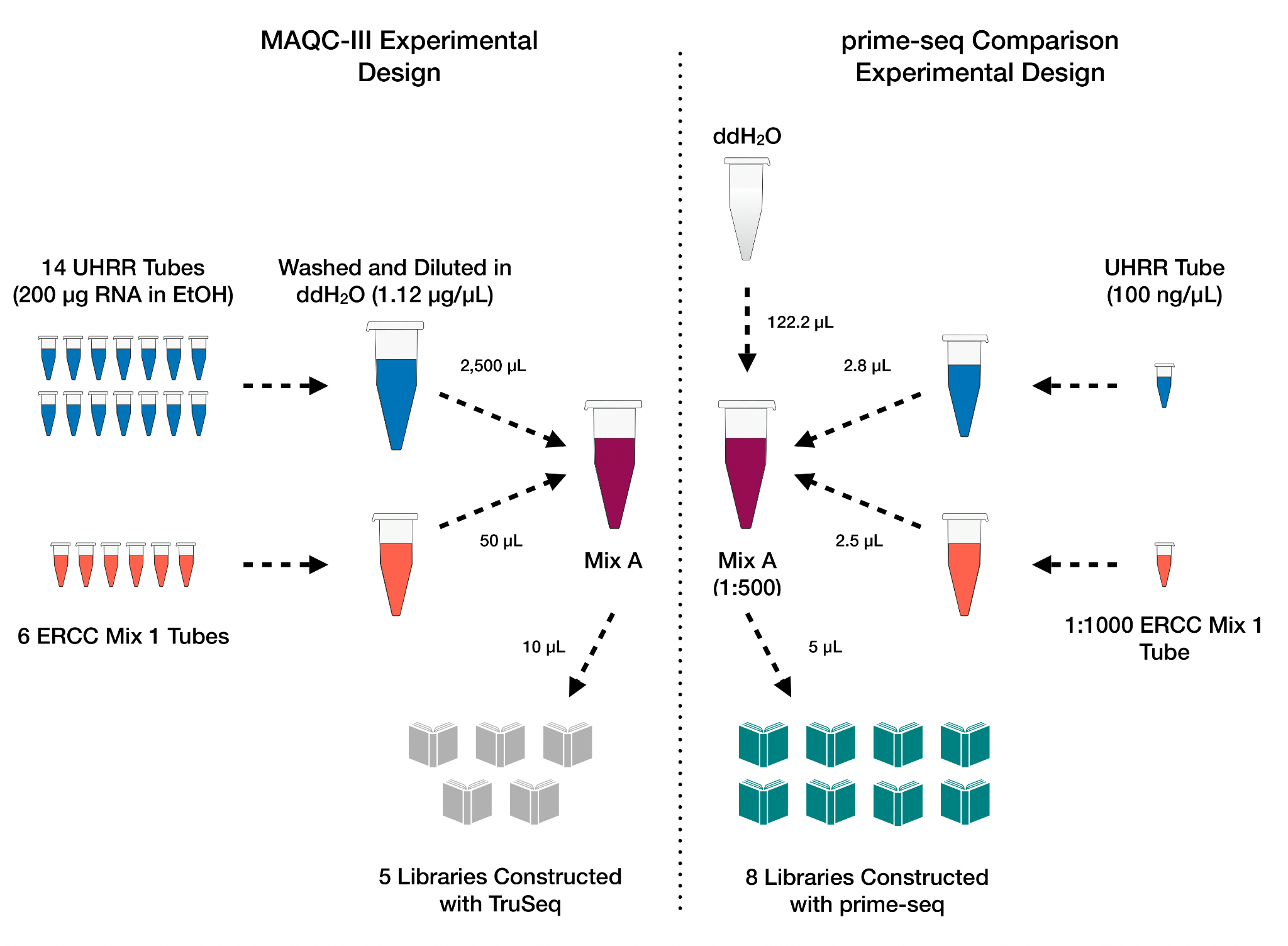


**Fig. S5. Experimental design comparing prime-seq to TruSeq data generated in the MAQC-III Study**. (Related to Figure 3) A 1:1000 concentration of Mix A, from the MAQC-III Study, was generated by mixing UHRR and ERCC Mix 1. From this, eight libraries were generated using prime-seq and compared to five TruSeq generated libraries.


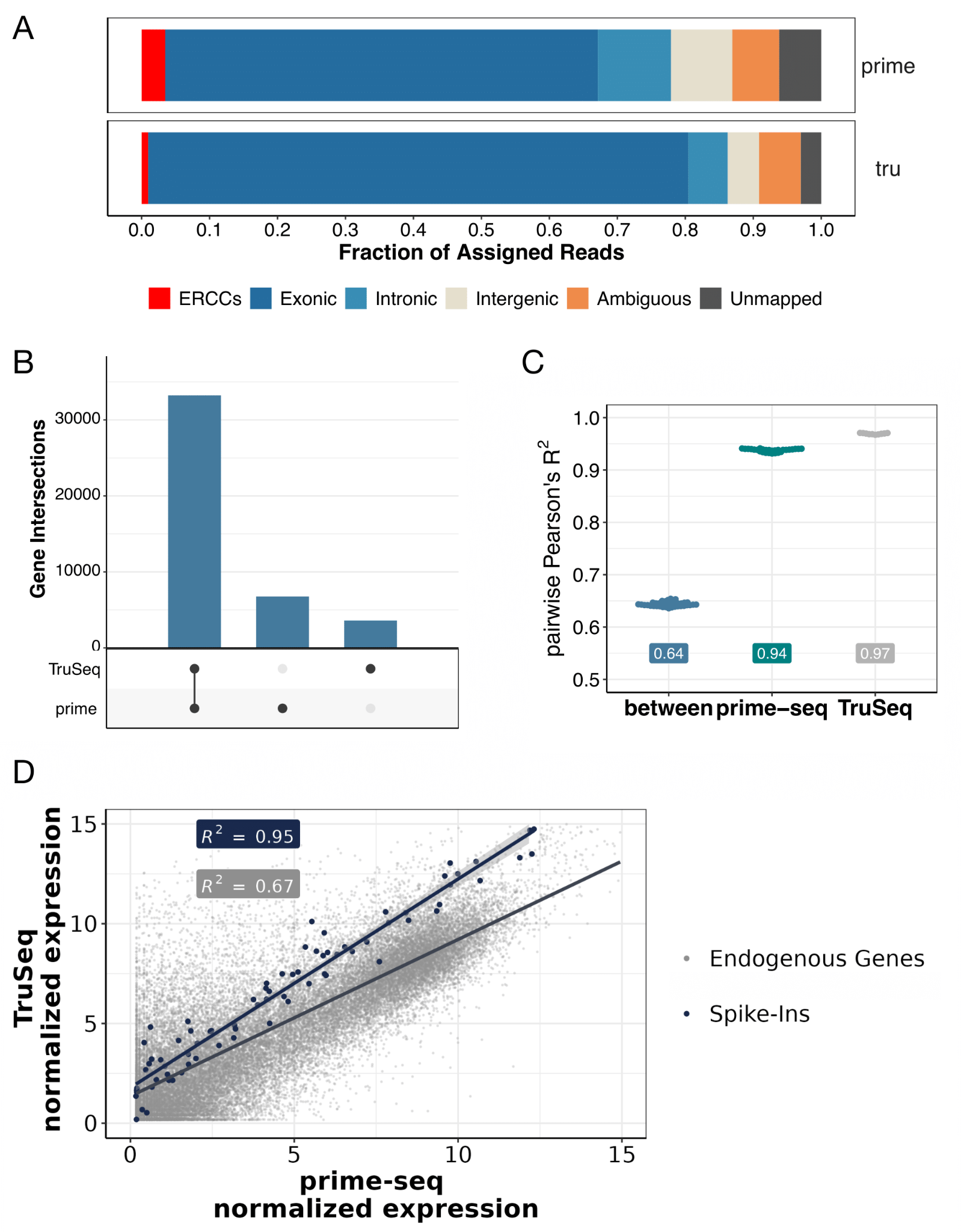


**Fig. S6. prime-seq and TruSeq have similar mapping, gene detection, and expression.**  (Related to Figure 3) (A) Feature distribution from prime-seq and TruSeq shows 78% and 85% of reads are exonic, intronic, and ERCCs, respectively. (B) TruSeq and prime-seq exhibit a strong overlap of detected genes (33,230), with 3,589 and 6,766 genes expressed only in TruSeq and prime-seq, respectively. (C) Coefficient of determination of two samples, either between (R^2^ = 0.64) or within methods (R^2^ = 0.94 for prime-seq and 0.97 for TruSeq). (D) Gene-wise scatterplot of prime-seq and TruSeq mean normalized expression showing decent correlation of endogenous genes (R^2^ = 0.67) and strong correlation of ERCC spike-in molecules (R^2^ = 0.95).

**
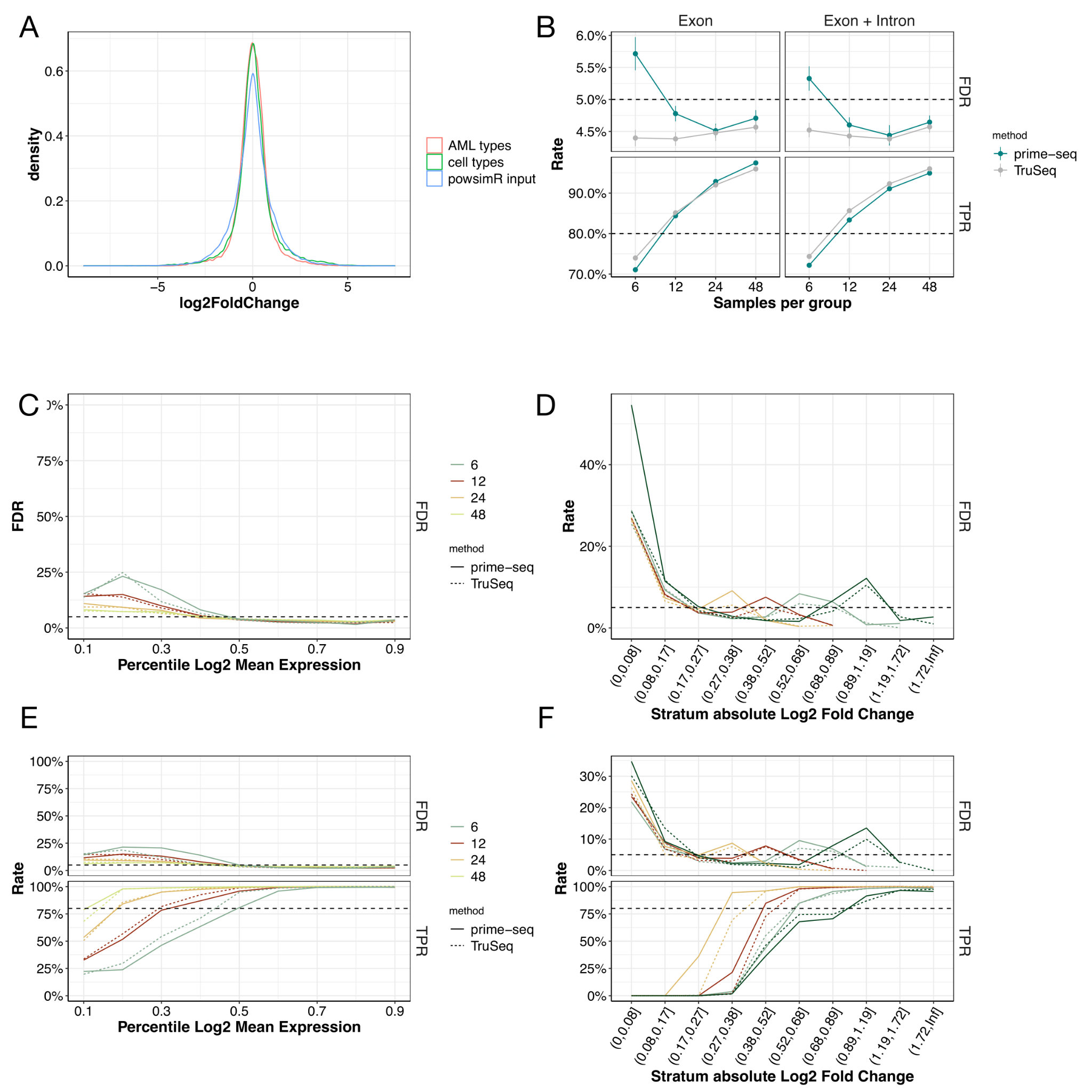
**

**Fig. S7. Power and FDR mostly depend on sample size and are similar between prime-seq and TruSeq.** (Related to Figure 3) (A) Log2 fold change distribution from the AML and NPC differentiation experiment (Figure 4) compared to the log2 fold change distribution used in powsimR for power analysis confirms that simulation settings match expected distributions. (B) Marginal power of prime-seq and TruSeq at differing samples per condition shows both methods perform similarly well, crossing the 80% threshold with roughly 12 samples both for exon plus intron and only exon counts. (C and D) FDR over different mean expression and log2 fold change strata (Related to 3F and 3G). (E and F) analogous to Figure 3F and 3G but including only Exonic counts; prime-seq and TruSeq exhibit similar TPR and FDR over different mean expression and log2 fold change strata. Filtering parameters: detected UMI ≥ 1, detected gene present in at least 25%.

**
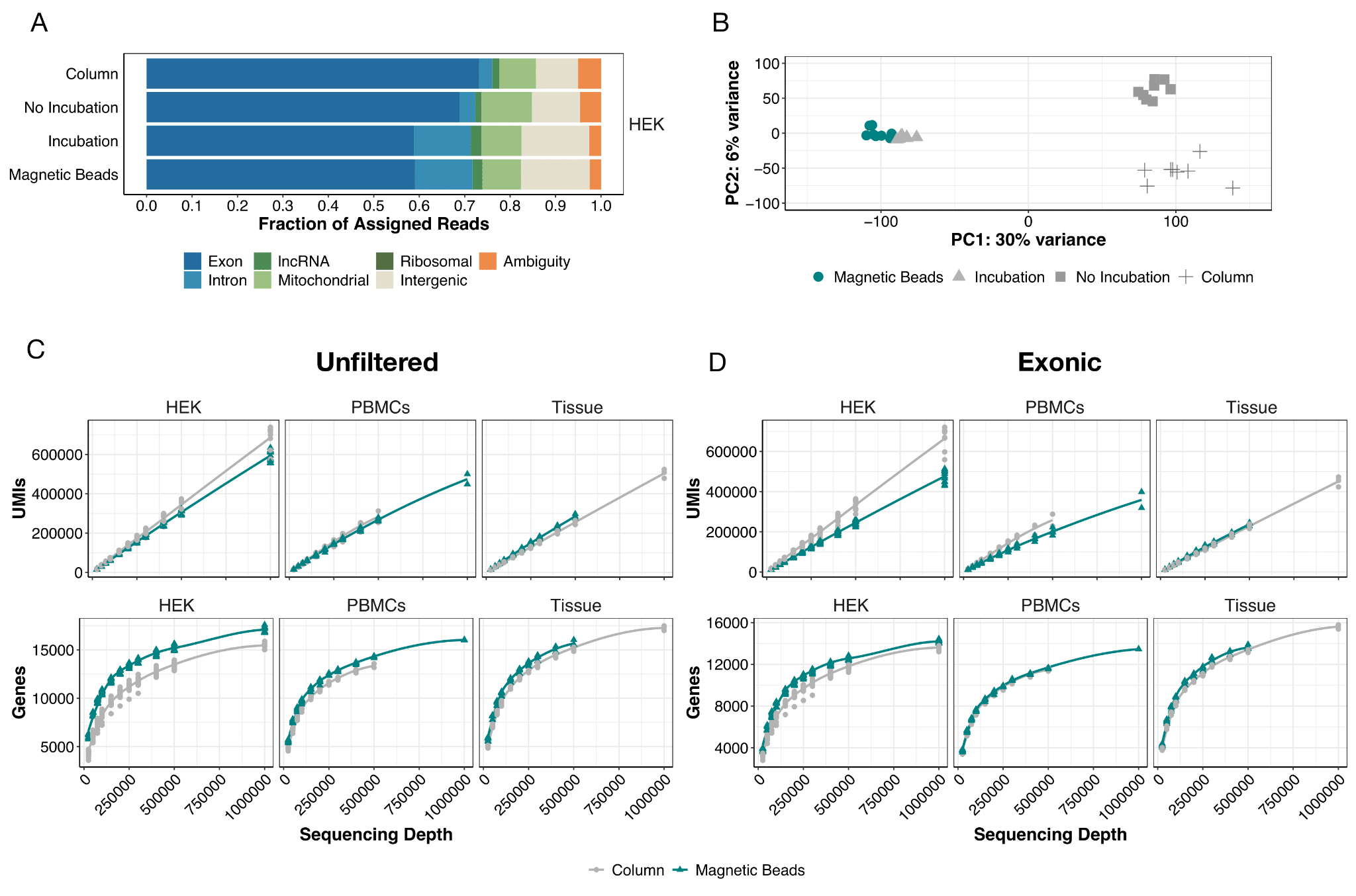
**

**Fig. S8. Performance of isolation methods is similar independent of prefiltering or usage of only Exon data.** (Related to Figure 4) (A) HEK293T cell samples were extracted using columns and magnetic beads, employing the standard prime-seq protocol (“Magnetic Beads”), as well as variant protocols without proteinase K digestion (“No Incubation”) and a proteinase K digestion control without enzyme (“Incubation”). All conditions had similar fractions of usable reads (all but intergenic and ambiguity), with an increase in intronic reads in “Incubation” and “Magnetic Beads” suggesting this increase is due to heat incubation. (B) Principal component analysis (PCA) of the 500 most variable genes shows the largest variable is heat incubation. (C and D) Analysis of detected UMIs and detected genes for unfiltered data and exonic only data shows that prime-seq using magnetic bead isolation is more sensitive in HEK cells and similarly sensitive in PBMCs and tissue compared to prime-seq using column isolation. Filtering parameters: detected UMI ≥ 1, detected gene present in at least 25% of samples and is protein coding.


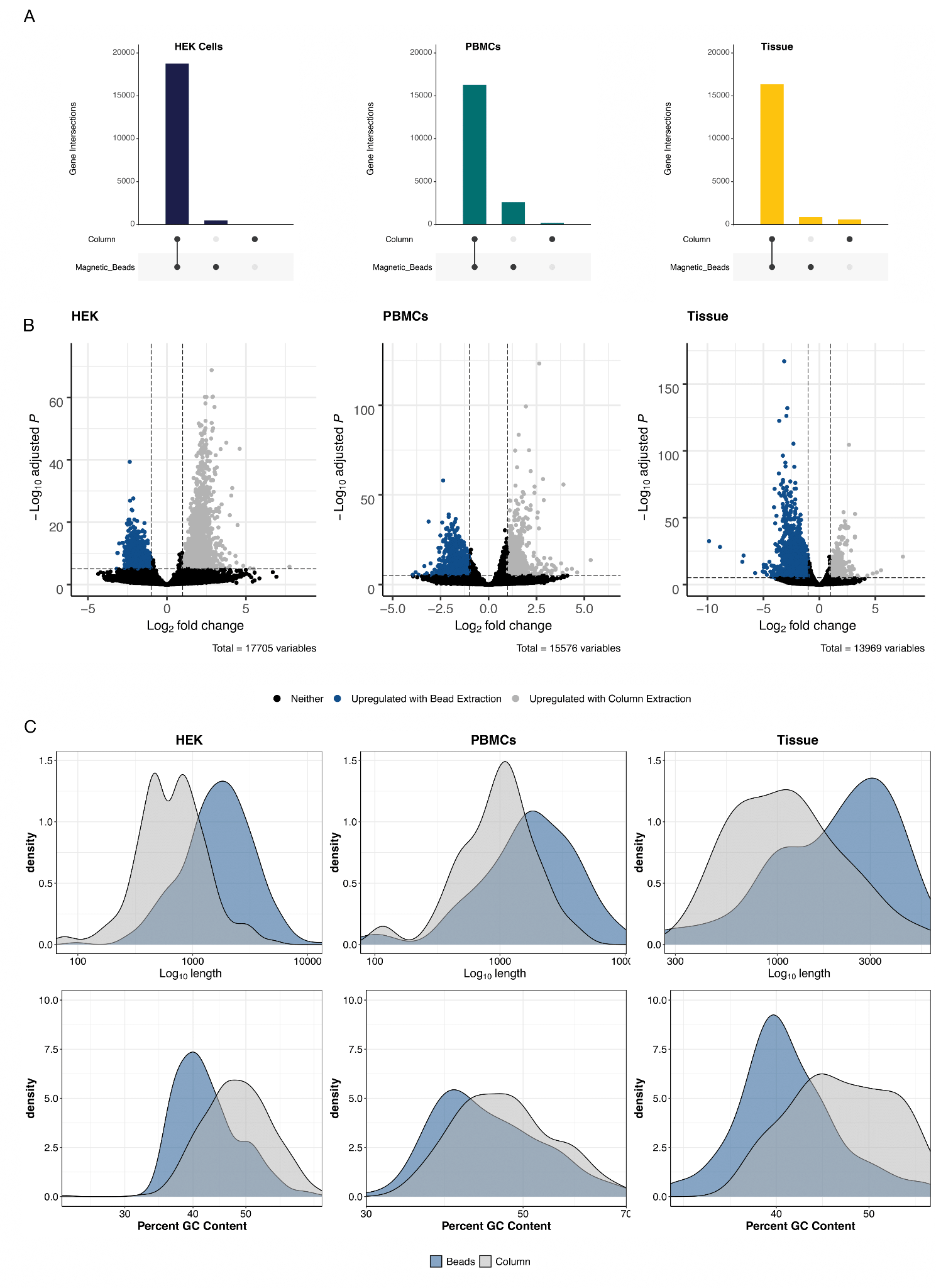


**Fig. S9. Most genes are detected independent of the extraction method used.** (Related to Figure 4) (A) Upset plots showing a strong overlap of detected genes between columns and magnetic beads. (B) Up- and down-regulated genes between column and bead-based RNA extractions (p>0.05, log_2_ FC > 2). (C) Density plots of the differentially expressed genes relative to length and GC content. Genes upregulated in columns tend to be longer with lower GC content.

**
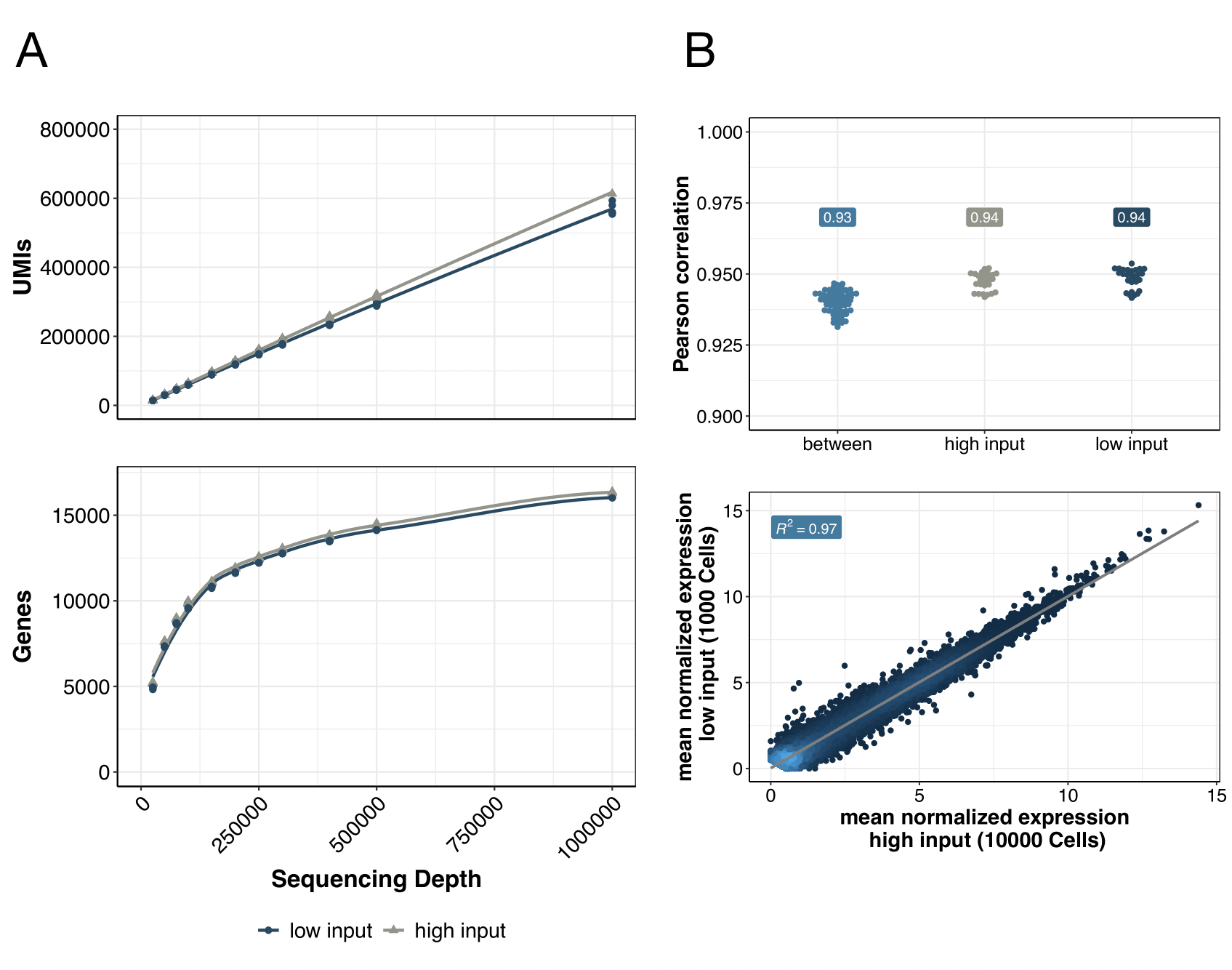
**

**Fig. S10. prime-seq performs equally well with high- and low-input samples.** (Related to Figure 5) (A) Sensitivity, measured in detected UMIs and genes, is similar between high input (10,000 HEK293T cells) and low input (1,000 HEK293T cells) conditions at various sequencing depths (filtering parameters: detected UMI ≥ 1, detected gene present in at least 25% of samples and is protein coding). (B) Additionally, Pearson's correlations between the high- and low-input conditions were high (pairwise comparison between: r = 0.93, pairwise comparison within: r = 0.94, and average normalized mean expression, R^2^ = 0.97).


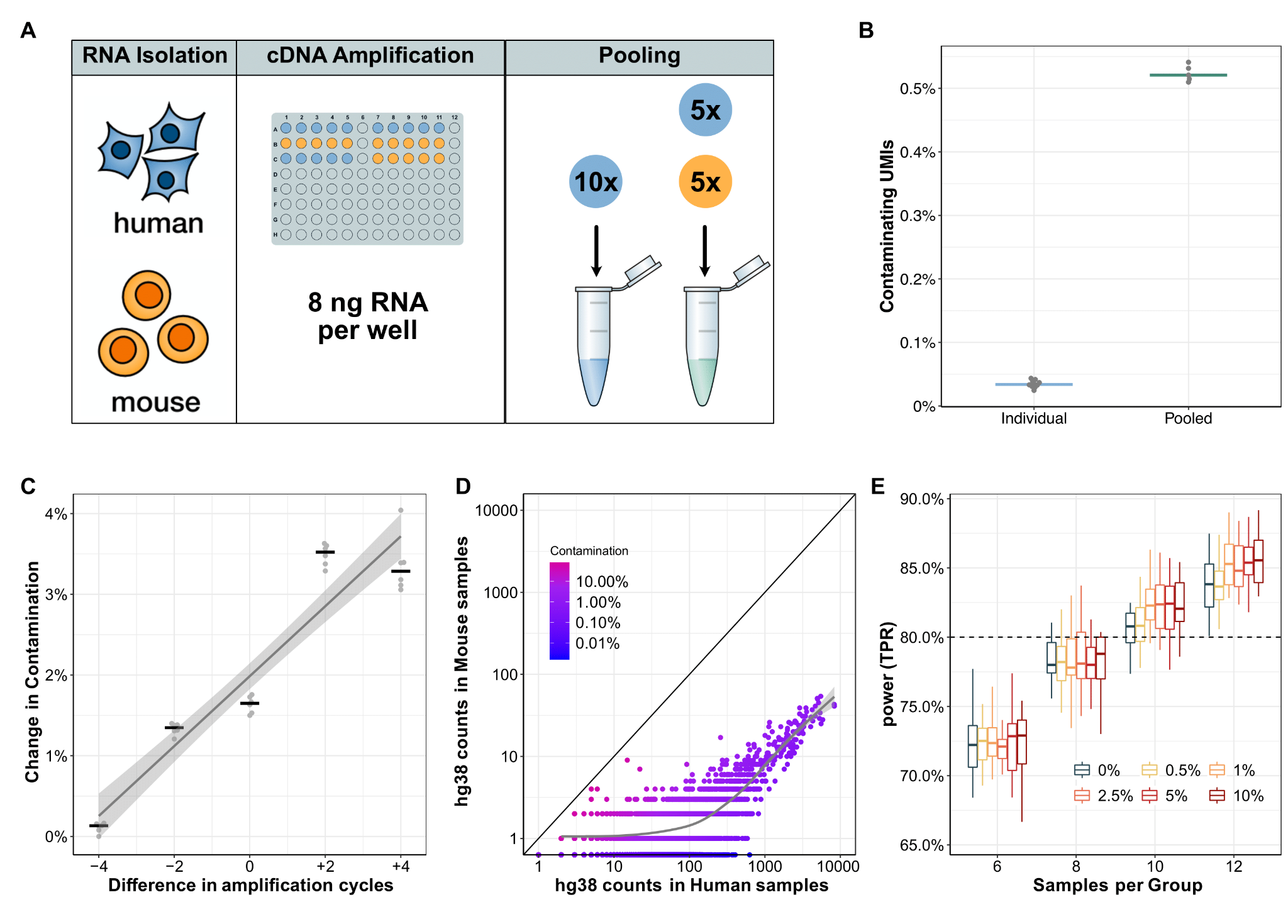


**Fig. S11. Cross-contamination levels are low, increase with additional cycles but do not impact power simulations.** (A) Experimental overview to detect cross-contamination. 1.RNA was isolated from hiPSCs and mESC; 2. cDNA amplification of 8ng RNA per well; 3. pooling of only human samples or mouse and human samples. (B) The percentage of contaminating UMIs (mapping best to the mouse genome) increases with pooling but is generally low median early pooling: 0.52%. (C) Impact of amplification cycles on cross-contamination. 0 corresponds to the condition shown in panel B, 13 cycles of pre-amplification for 96 ng of input RNA (8 ng per well). (D) Genewise contamination ranges from 0% to up to 10 % for lowly expressed genes. Contamination decreases with increasing expression levels. (E) Power simulation with different levels of computationally added contamination shows little impact on marginal TPR. An increase in the number of replicates leads to a small increase in power for highly contaminated conditions relative to no contamination.


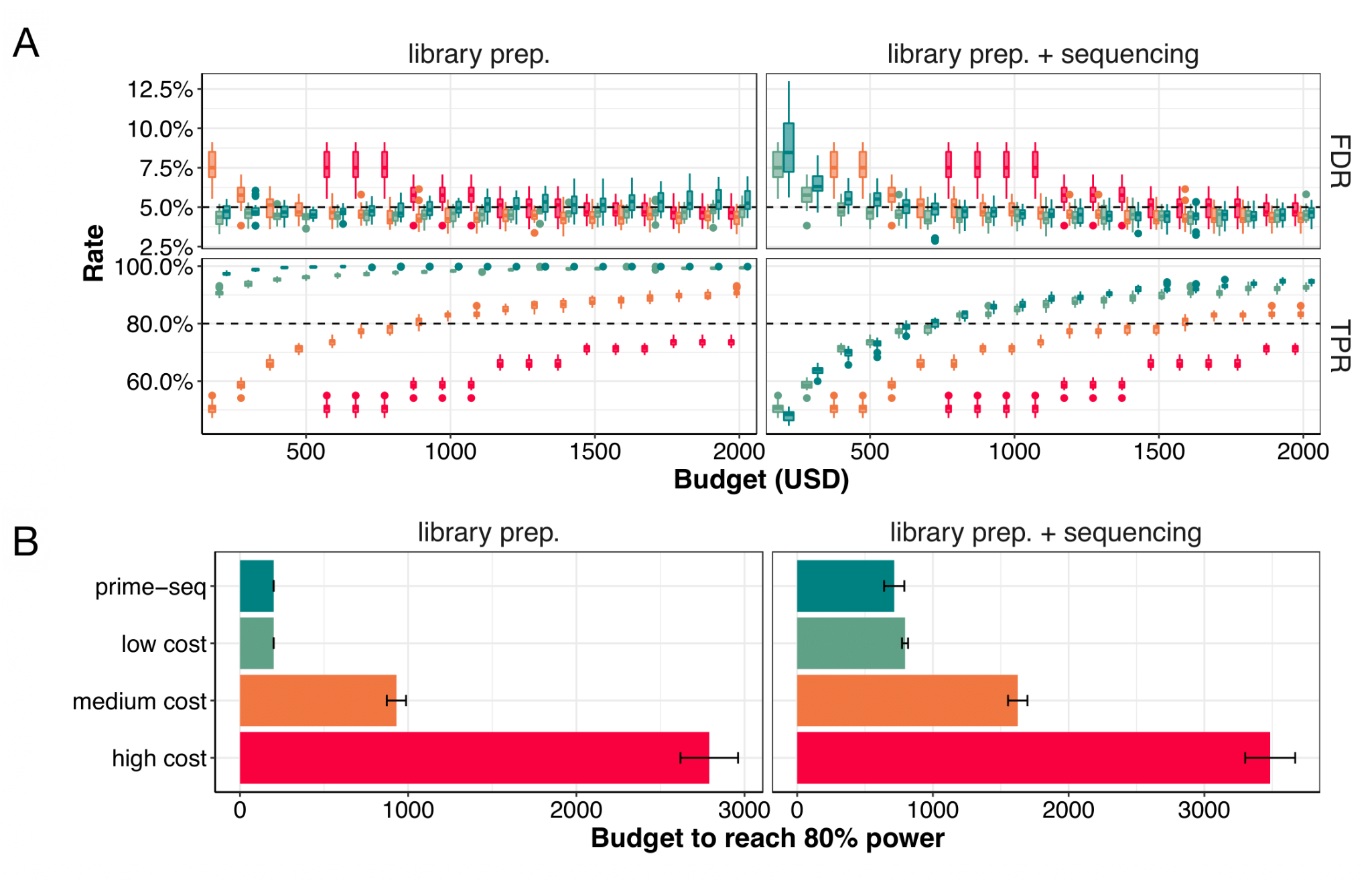


**Fig. S12. Power analysis shows prime-seq is able to reach 80% power earlier than less cost-efficient methods.** (Related to Figure 6) (A) True positive rate (TPR) and false discovery rates (FDR) corresponding to Figure 6B, but with more incremental values. (B) prime-seq crosses an 80% power threshold with $715 when sequencing costs are included compared to $795, $1,625, and $3,485 for low, middle, and high cost methods respectively (10 million reads used for analysis at a cost of $3.40 per 1 mio. reads).
