# Supplementary material for: Prime-seq, efficient and powerful bulk RNA sequencing
Source: Genome Biol. 2022 Mar 31;23:88. doi: 10.1186/s13059-022-02660-8 (PMC8969310; doi:10.1186/s13059-022-02660-8)
Supplement: Supplementary file 3 — Additional file 3: Supplemental Text. Magnetic Beads used in prime-seq. [file 13059_2022_2660_MOESM3_ESM.docx]

**Supplemental Text 1. Magnetic beads used in prime-seq**

Magnetic beads for nucleic acid isolation are a suitable alternative to column-based extraction, especially as they are more easily scalable and generally more cost efficient. These magnetic nanoparticles are coated to prevent oxidation and clumping, frequently with silica or a carboxyl coating as this provides an inert or negatively charged surface on the beads, respectively [50]. This allows for solid phase reversible immobilization (SPRI), that is the negatively charged nucleic acids can be precipitated out of solution, bind to the magnetic beads, be immobilized through the use of a magnet, washed, and then eluted without risk of irreversible binding. Additionally, carboxylated beads have carboxyl groups on the surface of the beads which form covalent bonds with nucleic acids in the presence of a crowding agent (i.e. polyethylene glycol) and high salt conditions [50]. These advantages have made carboxylated magnetic beads especially useful in isolating nucleic acids for high-throughput NGS applications.

For prime-seq, we use two different sets of beads, Carboxylated Sera-Mag SpeedBeads (GE Healthcare, now Cytiva) and SPRIselect (Beckman Coulter). The former are used in the RNA extraction step and the subsequent cleanup steps, whereas the latter are used only in the size selection steps during library preparation. Therefore, in the case of prime-seq, the Sera-Mag SpeedBeads are used to replace RNA extraction columns as well as nucleic acid concentrator columns, and the SPRISelect beads replace the gel excision and cleanup.

Numerous carboxylated magnetic beads exist for nucleic acid cleanups, with Ampure XP (Beckman Coulter) consistently used across many RNA-seq and NGS protocols. Additionally, the Ampure XP beads have worked well in our hands when used for standard nucleic acid cleanups. Within the prime-seq protocol, however, we specifically do not use the Ampure XP beads due to the cost factor involved. For example, a 24 sample prime-seq experiment would require 2.46 mL of Ampure XP beads and 2.7 mL of diluted Sera-Mag SpeedBeads prior to library preparation, which amounts to $203 and $2.25, respectively. The almost 90-fold lower cost makes it apparent that the Sera-Mag SpeedBeads are a better choice for many researchers. It could be possible that the Ampure XP beads yield a better nucleic acid recovery or provide more consistent performance, however, when tested we recover 80 % of input with the Sera-Mag SpeedBeads. Thus, even if there were slight improvements in performance, this would not outweigh the substantial increase in cost.

During library preparation, we are not only cleaning the solution to remove residual primers and salts, but are also specifically selecting for a range of fragment sizes (e.g. 300-800 bp). And, although both the Sera-Mag SpeedBeads and SPRIselect beads are carboxylated magnetic beads, the SPRIselect beads are validated for size selection properties ensuring consistent performance between lots. From our experience it is unclear if there are added advantages of using the SPRISelect beads for the library size selection, but as our Sera-Mag SpeedBead lot-to-lot verifications are not as rigorous as those performed by Beckman Coulter, and one only needs 70 µL of SPRISelect per prime-seq library, the increased cost of using SPRISelect for this portion of the protocol does not substantially alter the overall cost.
