# Supplementary material for: Prime-seq, efficient and powerful bulk RNA sequencing
Source: Genome Biol. 2022 Mar 31;23:88. doi: 10.1186/s13059-022-02660-8 (PMC8969310; doi:10.1186/s13059-022-02660-8)
Supplement: Supplementary file 8 — Additional file 8. Review history. [file 13059_2022_2660_MOESM8_ESM.docx]

Review History

**First round of review**

**Reviewer 1**

**Were you able to assess all statistics in the manuscript, including the appropriateness of statistical tests used?**

**No.**

**Were you able to directly test the methods?**

**No.**

**Comments to author:**

In this manuscript, Janjic et al reported Prime-seq, which is based on the 3' tagged scRNA-seq method incorporating the power of early barcoding for bulk RNA-sequencing. The protocol has been well utilized in nearly 22 publications during the past several years. The authors in particular focused on several aspects. First, they have integrated a direct lysis and RNA purification step, which further simplified the protocol. Second, they have analyzed intron sequences in RNA-seq, particularly using DNAse I treatment to show that the intronic reads were derived from RNA. Third, they have compared Prime-seq with TruSeq using standard RNA samples. Prime-seq performs similar to standard bulk RNA-seq while is more cost effective, especially when a lots of samples are needed for examination. The manuscript is well written. The authors have provided detailed protocol in their website and I expect that the method will be widely used. I have only a few questions.

1） The authors nicely showed that intronic reads are derived from RNA. The authors showed that that a large portion of genes (4000 out of 20000) were detected by intron only. Do these intronic reads always locate at the 3'gene regions, being the last intron? How about the expression levels of these intron-only genes, comparing with the exon-only and both genes? Do the authors interpret these intron-only genes as pre-mRNAs?

2）Figure 2B. It looks that with the DNA ratio increasing from 50% to 75%, the contamination ratio did not increase in proportion. Can the authors explain this? The authors found that ~4% DNA contamination if gDNA is not digested. Do these contaminated DNA sequences enrich any region, for example, transposons? Since standard scRNA-seq do not digest gDNA, this information may help improvement of scRNA data analysis.

3）Figure 3E. It is surprising that highly expressed genes have high variations, both for prime-seq and Truseq. Can the authors explain it?

4）Bead-based extraction. The use of a direct lysis process is an important improvement of Prime-seq as it increases cost-efficiency of library production. The authors used SpeedBeads from Sigma-Aldrich. Have the authors tried other brands of types of beads? Will the bead brand or type affect the results?

5）As the authors have discussed, cross-contamination is an important concern especially for an early barcoding method as prime-seq. The authors have claimed that this is not an issue in prime-seq and showed a brief result in the discussion part. However, since this is important, it will be better that the authors describe more details of the experiment and display the results as supplementary figures. For example, the authors said that "low rates of cross-contamination when samples were pooled as RNA (0.59%), cDNA (0.76%), or libraries (0.83%)". Since the RNA is the first step, it should give the highest contamination rates. So as I understand, the net contamination rate is to add up three values, as 2.18%? Also, will the contamination ratio increase with PCR cycles?

**Reviewer 2**

**Were you able to assess all statistics in the manuscript, including the appropriateness of statistical tests used?**

**No.**

**Were you able to directly test the methods?**

No.

**Comments to author:**

For a long time, many gene expression profiling studies have used an insufficient number of replicates, despite that biostatisticians have proved this low number of replicates are underpowered for identifying differentially expressed genes. To address this critical issue, Genome Biology has recently published two relative methods, BRB-seq and Decode-seq. This work developed another method, Prime-seq. It made several key improvements in the experimental and computational procedure, including direct RNA isolation by beads, and using intronic reads for expression level estimation. Eventually, the authors showed that this method performed equivalent to TruSeq in terms of library complexity, measurement accuracy, and statistical power. Overall, the method is well documented, the conclusions are supported by the data. Prime-seq would be another tool for a wide range of experimental biologists to address the underpower issue in DE analysis. Therefore, I would like to support publishing this paper on Genome Biology.

A few minor questions:

1. In Fig3 comparisions, are intronic reads from TruSeq used in the calculation? what are R^2 between expression levels of all genes measured by TruSeq and Prime-seq, and among replicates of two methods?

2. The github link is not accessible.

**Authors Response**

**Point-by-point responses to the reviewers’ comments:**

Reviewer #1:

In this manuscript, Janjic et al reported Prime-seq, which is based on the 3' tagged scRNA-seq method incorporating the power of early barcoding for bulk RNA-sequencing. The protocol has been well utilized in nearly 22 publications during the past several years. The authors in particular focused on several aspects. First, they have integrated a direct lysis and RNA purification step, which further simplified the protocol. Second, they have analyzed intron sequences in RNA-seq, particularly using DNAse I treatment to show that the intronic reads were derived from RNA. Third, they have compared Prime-seq with TruSeq using standard RNA samples. Prime-seq performs similar to standard bulk RNA-seq while is more cost effective, especially when a lots of samples are needed for examination. The manuscript is well written. The authors have provided detailed protocol in their website and I expect that the method will be widely used. I have only a few questions.

*Response: We thank the reviewer for this positive assessment.*

1） The authors nicely showed that intronic reads are derived from RNA. The authors showed that that a large portion of genes (4000 out of 20000) were detected by intron only. Do these intronic reads always locate at the 3'gene regions, being the last intron? How about the expression levels of these intron-only genes, comparing with the exon-only and both genes? Do the authors interpret these intron-only genes as pre-mRNAs?

*Response: We have now added an analysis of the location of intronic and exonic reads as well their expression levels and correlation with exon counts in the new Supplementary Figure S4 and added this to the appropriate Results section (Intronic reads are derived from RNA).*

*Briefly, we find an almost as strong 3’ bias for intronic reads as for exonic reads and a slightly lower expression level, compatible with a mix of internal and poly(A) priming of pre-mRNA. We now make clearer that we interpret intronic reads as pre-mRNA in line with previous publications.*

2）Figure 2B. It looks that with the DNA ratio increasing from 50% to 75%, the contamination ratio did not increase in proportion. Can the authors explain this? The authors found that ~4% DNA contamination if gDNA is not digested. Do these contaminated DNA sequences enrich any region, for example, transposons? Since standard scRNA-seq do not digest gDNA, this information may help improvement of scRNA data analysis.

*Response: We think the previous Figure was a bit misleading and confusing and we have revised it now with the response variable (% genomic reads) on the y-axis. The plotting of individual data points now also makes clear that the measurement errors are too large to conclude a deviation from a linear increase.*

*We also now mention the relevance of these findings for scRNA-seq data by saying that “..suggesting that also for gDNA containing samples (e.g. single cells) the impact of genomic reads on expression levels is likely small.” We did not see any obvious enrichment of particular sequences like transposons that would be informative for scRNA-seq data and decided to not include such an analysis as we feel is also too far from the papers main topic.*

3）Figure 3E. It is surprising that highly expressed genes have high variations, both for prime-seq and Truseq. Can the authors explain it?

*Response: This Figure is actually misleading as the visually dominating increase in variation at very high expression levels depends on very few (10-15) very highly expressed genes (compare Figure 3D). The LOESS fitting in combination with the “raw” estimation of the values by methods of moments we use is then not very suitable. This is no problem for our argumentation (as we anyway continue with power simulations to interpret the variation), but it is visually misleading. We solved this by combining Figure 3D and 3E and restricting the plot to expression levels where estimation of the loess fit was trustworthy. We exchanged the Figure and adapted the text accordingly.*

4）Bead-based extraction. The use of a direct lysis process is an important improvement of Prime-seq as it increases cost-efficiency of library production. The authors used SpeedBeads from Sigma-Aldrich. Have the authors tried other brands of types of beads? Will the bead brand or type affect the results?

*Response: This is an interesting point. In short we use the cheapest beads available and other brands are either the very same beads from General Electric (GE Health) or they are much more expensive. We have now added a Supplemental Note discussing the choice of beads in more depth (Supplemental Text 1).*

5）As the authors have discussed, cross-contamination is an important concern especially for an early barcoding method as prime-seq. The authors have claimed that this is not an issue in prime-seq and showed a brief result in the discussion part. However, since this is important, it will be better that the authors describe more details of the experiment and display the results as supplementary figures. For example, the authors said that "low rates of cross-contamination when samples were pooled as RNA (0.59%), cDNA (0.76%), or libraries (0.83%)". Since the RNA is the first step, it should give the highest contamination rates. So as I understand, the net contamination rate is to add up three values, as 2.18%? Also, will the contamination ratio increase with PCR cycles?

*Response: We fully agree with this point and have now done additional, better controlled experiments and summarized them in a new Results section (Barcode swapping in prime seq is low).*

*Briefly, we now compare the same number of libraries (N=10) of mouse and human RNA prepared separately or pooled after first strand synthesis. We find ~0,5% swapped barcodes (“contamination”). Note that this is comparable to our previous estimate (0.59%) and that cDNA and library contamination was higher, probably due to variations among library amplifications and/or due to previous differences in the total amount of input used.*

*We also compared the number of PCR cycles and find- as expected - that the amount of swapped barcodes does increase with additional cycles. Using simulations, we show that even up to 10% of swapped barcodes have only very little impact on power. We also now do discuss more explicitly that barcode swapping needs to be considered when interpreting qualitative (presence/absence) gene expression levels and that it needs to be better investigated and controlled as it has been discussed so far only in the context of scRNA-seq.*

Reviewer #2:

For a long time, many gene expression profiling studies have used an insufficient number of replicates, despite that biostatisticians have proved this low number of replicates are underpowered for identifying differentially expressed genes. To address this critical issue, Genome Biology has recently published two relative methods, BRB-seq and Decode-seq. This work developed another method, Prime-seq. It made several key improvements in the experimental and computational procedure, including direct RNA isolation by beads, and using intronic reads for expression level estimation. Eventually, the authors showed that this method performed equivalent to TruSeq in terms of library complexity, measurement accuracy, and statistical power. Overall, the method is well documented, the conclusions are supported by the data. Prime-seq would be another tool for a wide range of experimental biologists to address the underpower issue in DE analysis. Therefore, I would like to support publishing this paper on Genome Biology.

*Response: We thank the reviewer for this positive assessment.*

A few minor questions:

1. In Fig3 comparisions, are intronic reads from TruSeq used in the calculation? what are R^2 between expression levels of all genes measured by TruSeq and Prime-seq, and among replicates of two methods?

*Response: Yes, for both intronic reads are used and we write this now also in the figure legend.*

*We have also now included a more detailed comparison of prime-seq and TruSeq expression levels including R^2 values that are high within and low between methods (Figure S5 B,C,D). Note that the artificial ERCC have a rather high correlation between methods (R^2=0.95), in contrast to the endogenous genes (R^2=0.67). We attribute this to the controlled in vitro generation of ERCC molecules in contrast to the less controlled in vivo mixture of 10 cancer cell lines of the UHR RNA that has apparently changed considerably since the MAQCIII study. Note also that the power comparisons just assume an equally complex and distributed RNA population and in this respect the UHRR sample we use and the UHRR sample that was used by the MAQCIII study are well comparable (Fig. 3 A,B & D).*

2. The github link is not accessible.

*Response: Unfortunately, the repository was not set to public. We fixed this issue now and are sorry for this inconvenience. We have additionally updated the github repository and made it available through zenodo.*

**Second round of review**

**Reviewer 1**

My questions have been answered.
